# TWAS revealed significant causal loci for milk production and its composition in Murrah buffaloes

**DOI:** 10.1038/s41598-023-49767-x

**Published:** 2023-12-16

**Authors:** Supriya Chhotaray, Vikas Vohra, Vishakha Uttam, Ameya Santhosh, Punjika Saxena, Rajesh Kumar Gahlyan, Gopal Gowane

**Affiliations:** 1 Division of Animal Genetics and Breeding, ICAR-Central Institute for Research on Buffaloes, Hisar, Haryana 125001 India; 2 Animal Genetics and Breeding Division, ICAR-National Dairy Research Institute, Karnal, Haryana 132001 India

**Keywords:** Animal breeding, Eukaryote, Functional genomics, Gene expression, Gene regulation, Genetic association study, Genetic markers, Genome, Genomics, Population genetics, Quantitative trait, Sequencing

## Abstract

Milk yield is the most complex trait in dairy animals, and mapping all causal variants even with smallest effect sizes has been difficult with the genome-wide association study (GWAS) sample sizes available in geographical regions with small livestock holdings such as Indian sub-continent. However, Transcriptome-wide association studies (TWAS) could serve as an alternate for fine mapping of expression quantitative trait loci (eQTLs). This is a maiden attempt to identify milk production and its composition related genes using TWAS in Murrah buffaloes (*Bubalus bubalis*). TWAS was conducted on a test (*N* = 136) set of Murrah buffaloes genotyped through ddRAD sequencing. Their gene expression level was predicted using reference (*N* = 8) animals having both genotype and mammary epithelial cell (MEC) transcriptome information. Gene expression prediction was performed using Elastic-Net and Dirichlet Process Regression (DPR) model with fivefold cross-validation and without any cross-validation. DPR model without cross-validation predicted 80.92% of the total genes in the test group of Murrah buffaloes which was highest compared to other methods. TWAS in test individuals based on predicted gene expression, identified a significant association of one unique gene for Fat%, and two for SNF% at Bonferroni corrected threshold. The false discovery rates (FDR) corrected P-values of the top ten SNPs identified through GWAS were comparatively higher than TWAS. Gene ontology of TWAS-identified genes was performed to understand the function of these genes, it was revealed that milk production and composition genes were mainly involved in Relaxin, AMPK, and JAK-STAT signaling pathway, along with CCRI, and several key metabolic processes. The present study indicates that TWAS offers a lower false discovery rate and higher significant hits than GWAS for milk production and its composition traits. Hence, it is concluded that TWAS can be effectively used to identify genes and cis-SNPs in a population, which can be used for fabricating a low-density genomic chip for predicting milk production in Murrah buffaloes.

## Introduction

The transcriptome-wide association study (TWAS) is an emerging gene-based association with phenotype that leverages the fine mapping of expression quantitative trait loci (eQTLs) and identification of causal genes with higher power than conventional genome-wide association studies (GWASes). TWAS uses the predicted gene expression levels as predictor variables affecting the phenotype variance unlike the GWAS that uses genotypes as the causal variables^1^. In the era of multi-“Omics”, integration of genome-wide SNP genotypes and transcriptome information to achieve fine mapping of causal variants for complex traits has been an essential step. Though its importance is evident, yet fewer studies have been conducted for complex economic traits in dairy animals. Majority of such studies focusses on identification of eQTLs based on GWAS^2,3^ and^4^ and post-GWA study on differentially expressed genes (DEGs) and weighted gene co-expression network analysis (WGCNA)^5^. However, the majority of GWAS-hit loci lie in non-coding regions^6^ and even though they might play a role in gene expression regulation, its physiological perspective is unclear. In dairy animals, most of the variants contributing to complex lactation traits have not yet been identified due to a limit on detection of true positives through GWAS in small sample sizes. Since the conception of TWAS^7^, several studies have been conducted in humans for psoriasis^8^, Depression^9^, hematological traits^10^, and Alzheimer’s disease^11^ etc. Apart from that, TWAS has been performed in maize^12^, and in pigs for meat quality traits^13^.

Several studies in humans indicate the superiority of TWAS power over GWAS when expression heritability (h_e_^2^) varies between 0.04 and 0.2 and proportion of variance explained by causal cis-SNPs is low^1,14^. The advantage of TWAS lies in its statistical power in identifying a causal gene with much lesser sample size than GWAS and its robustness to incorporate both individual and summary statistics of GWASes^15^. Once the gene expression prediction model is developed, it can fit across studies and tissues that further increase the prediction accuracy. Riverine buffalo (*Bubalus bubalis*) being a major dairy animal with ~ 45% contribution to the national milk production in India, still remains aloof from the genomic research on its genetic architecture^4^. Integrating transcriptome and genome-wide SNP information shall help in delineating the causal genes for lactation traits in one of the major dairy breed of buffaloes i.e., Murrah. The present study aims at devising a suitable gene expression prediction model based on SNP genotypes and to associate the predicted expression levels with various lactation traits such as 305 days milk yield, peak yield, wet average and milk composition traits like fat and SNF% in Murrah buffalo. The scope of this study is identification of causal genes and cis-SNPs with higher effect sizes on these causal genes for the studied traits such that they can further be used in genomic selection and improvement programs.

## Materials and methods

Lactation records of 144 randomly selected female Murrah buffaloes from Livestock Research Centre (LRC), National Dairy Resaerch Insitute (NDRI), Karnal, India (29.68°N and 76.99°E) were collected for the present study. 1st lactation records of 305 days milk yield (305 DMY), peak yield (PY), wet average (WA), fat percentage (fat%), Solid-not-fat percentage (SNF%), birth weight (bwt) in kg, and age at first calving (AFC) in months for 144 selected buffaloes which had completed their first lactation with a standard lactation length of 305 days or more were recorded. Generally, the animals are stall-fed and as let-down ration, 0.25 kg of additional concentrate is given at the time of milking. Green fodder and other roughages are provided in ad-libitum. All the buffaloes are exclusively stall fed in open paddocks at the LRC, NDRI.

The animal study was reviewed and approved by the ICAR-National Dairy Research Institute (IAEC). All experiments were performed in accordance with the guidelines and regulations of IAEC, ICAR-NDRI.

### Generation of genotype information

Blood sample from 144 randomly selected animals were collected aseptically and DNA was isolated via Phenol–Chloroform method following protocol of Sambrook and Russell^16^. Quality of DNA was checked using agarose gel electrophoresis and quantity was assessed using Qubit 4.0 fluorometer. DNA double digestion with *SphI* and *MluCI* restriction enzymes was carried out for standard restriction-associated DNA (RAD) protocol as described by^17^. Standard Illumina read multiplexing protocol was followed with adapters (P1 and P2). After adapter ligation and size selection, samples were sequenced on Illumina Hi-seq 2000 platform and 150 bp paired end reads were generated with ~ 1X coverage. Index and sequence dictionary files for reference genome retrieved from NCBI website were created using the Burrows–Wheeler algorithm (BWA)^18^ and PicardTools, respectively. The quality of paired-end raw FASTQ files generated after sequencing, was checked using FastQC^19^. Adapters were marked and trimmed using bbmap^20^. The BWA-MEM algorithm was used to align the trimmed FASTQ sequences with the reference genome. Aligned files were coordinate-sorted, and duplicate reads were removed. Read group identifiers were updated using PicardTools. The quality of aligned BAM files was checked using qualimap^21^. Variants were called using bcftools-mpileup^22^. This variant calling pipeline was previously standardized in our laboratory^23^ and two sets of variant calling were performed. Set-I variants were called based on the latest Murrah buffalo reference genome GCF_019923935.1_NDDB_SH_1_genomic.fna (https://www.ncbi.nlm.nih.gov/assembly/GCF_019923935.1) and variants were retained for further training of the dataset to predict eQTL weights and individual level transcriptome wide association study. A second set (Set-II) of variants were called based on the Mediterranean buffalo reference genomeGCF_003121395.1_ASM312139v1_genomic.fna(https://www.ncbi.nlm.nih.gov/assembly/GCF_003121395.1) for GWAS.

### Quality control (QC) check of variants for downstream analysis

Set-I SNPs were further QC checked using PLINK v1.9^24^ and all the indels were removed from further analysis. Only biallelic variant sites on autosomes and X chromosome having a genotype rate > 95% were retained. Variants passing the threshold of Hardy–Weinberg equilibrium test at p < 0.0001 and minor allele threshold of 0.01 were retained for TWAS.

Set-II SNPs were also QC checked using PLINK v1.9 with thresholds of genotyping rate > 95%, linkage disequilibrium (LD) in terms of r^2^ < 0.8, not deviating from HWE at p < 0.0001, and with MAF > 0.05. Only autosomal and X chromosomal SNPs were retained for GWAS.

### Genome-wide association study (GWAS)

GWAS was conducted with a set of 39,019 QC passed Set-II SNPs, for the 1st lactation 305 days milk yield, peak yield, wet average, fat% and SNF% using the true phenotypes for all the 144 individuals that had completed the 1st lactation with a standard 305 days of lactation. Genome-wide identity-by-state (IBS) for all pairs of individuals was checked. Multidimensional scaling (MDS) based on SNP information was done to check for the presence of any population stratification and was corrected by incorporating the first two MDS components as covariates in the model for GWAS. Birth weight (bwt) and age at first calving in months (AFC) were also included as covariates in the model. A genome-wide scan for significant SNPs considering only additive effects was accomplished through a simple regression model using PLINK v1.9 as described by Marees et al.^25^, where residuals were assumed to be normally and independently distributed. A linear regression model was fitted for determining the association between SNPs and continuous traits^26^. The threshold for genome-wide significance was determined by correcting the *P*-values of the SNP association test with Bonferroni’s correction and was 1.28 $\times$ 10^–6^. *P*-values of the top ten SNPs of each GWAS traits was corrected for Benjamini–Hochberg’s false discovery rate (FDR) at 5% levels^27^ using the “R” package fuzzySim v3.0^28^ and is given in the supplementary document. The results were plotted as Manhattan plots and Q-Q plots using the “qqman” package of R.

Linear regression model used for GWAS:

$$y = \, \upbeta_{0} + x*\upbeta_{{1}} + {\text{C}}_{{1}} *\upbeta_{{2}} + {\text{C}}_{{2}} *\upbeta {3}_{{}} + {\text{AFC}}*\upbeta_{{4}} + {\text{ bwt}}*\upbeta_{{5}} + {\text{ e}}$$

where, *y* = 1st lactation 305 days milk yield, *x* = additive effect of SNPs, C_1_ = first component of MDS, C_2_ = second component of MDS, AFC = Age at first calving in months, bwt = birth weight of the animal (in kgs), β_0_ = intercept term, β_1_ = regression coefficient representing the strength of association between SNP *x* and trait *y*, β_2_ = regression coefficient of C_1_, β_3_ = regression coefficient of C_2_, β_4_ = regression coefficient of AFC, β_5_ = regression coefficient of bwt, and *e* = residuals or noise not explained by SNPs. Eldawy et al.^29^ reported significant effect of body weight at birth and AFC on the reproduction and production performances in dairy buffaloes. Hence, these two variables were considered as important covariates for the GWAS and TWAS models.

### Generation of gene expression information

For integrating transcriptomic information to find the underlying gene significantly contributing to the expression of complex lactational traits, 8 animals in the 2nd parity in a mid-lactation stage in the winter season were selected as reference animals for the TWAS having both genotype and phenotype data. The animals from the institute herd that had calved during the autumn and reached the peak lactation stage during the winter were selected as reference animals. To maintain the homogeneity, milk samples were collected from the animals in the mid lactation where animals attend their peak during the beginning weeks of this stage, which comes during the winter in the present study. The 1st lactation average in the herd was 2122.5 ± 286 kg. The reference animals were divided into two groups; above average + 1σ were considered high yielders (*N* = 5; > 2400 kg/lactation) while animals below average-1σ were treated as low yielders (*N* = 3; < 1800 kg/lactation) in the present study. Approximately, 150–200 ml of milk was collected aseptically in Diethyl pyrocarbonate (DEPC) treated tubes. RNA isolation was performed under sterile conditions in lab. RNA isolation was done following a hybrid protocol^30^ from the fat layer of the milk. Extracted RNA quantity was checked on Qubit 4.0 fluorometer and library was prepared for good quality samples with high RIN (> 6.5) values. Sequencing was performed using Illumina Novoseq 6000 platform. RNAseq data analysis was performed following the standard Galaxy workflow^31^. Adapters were trimmed using cutadapt v3.7 allowing a maximum error rate of 0.1. Trimmed RNAseq fastq files were aligned to the reference genome GCF_019923935.1_NDDB_SH_1_genomic.fna using BWA-MEM algorithm. Aligned BAM files were sorted by chromosomal coordinates and other post-alignment cleaning processes such as deduplication, and sample information update were completed using picardtools. Qualimap–RNAseqQC and BAMQC v2.2.2-dev were used for checking the quality of aligned BAM files. Feature counts were generated using featureCounts v2.0.1. assuming reads are forward stranded. Fragments were counted for the paired-end data only if both the reads were aligned after removing chimeric fragments. rLog normalized gene expression levels from DESeq2 v2.11.40.7 were obtained after correcting for the “production level” i.e., high and low yields and “batch of sample collection”.

### Transcriptome-wide association study

To perform a two stage TWAS, first gene expression imputation model was designed for estimating the cis-eQTL effect sizes from a training sample (*N* = 8) for which both genotype and transcriptome data are available. The model suggested by Nagpal et al.^14^ was employed which is as follows:

1 $${\text{E}}_{{\text{g}}} = X_{{{\text{train}}}} {\text{w }} + \varepsilon , \, \varepsilon \, \sim N\left( {0, \, \upsigma_{\varepsilon }^{{2}} I} \right)$$

where, E_g_: denotes the log normalized gene expression levels (after corrections for confounding factors such as production levels and sampling batch) for gene g. *X*_train_: denotes the genotype matrix for all cis-genotypes (encoded as the number of minor alleles present 1 MB of the gene; [− 1 MB—Gene_start—Gene_end— + 1 MB]). w: denotes the corresponding cis-eQTL effect-size vector, and ε: denotes the error term.

The gene expression levels GReX (genetically regulated gene expression) of the test samples (*N* = 136) were imputed with the assumption of following model:

2 $$G\hat{R}eX = X_{{{\text{test}}}} \hat{w}$$

Given the predicted eQTL effect size estimates $\hat{w}$ from the training data in Eq. (1), *GReX* was imputed by the Eq. (2) where *X*_test_ is the genotype matrix containing cis-SNP data for the test dataset.

**Figure 1:**
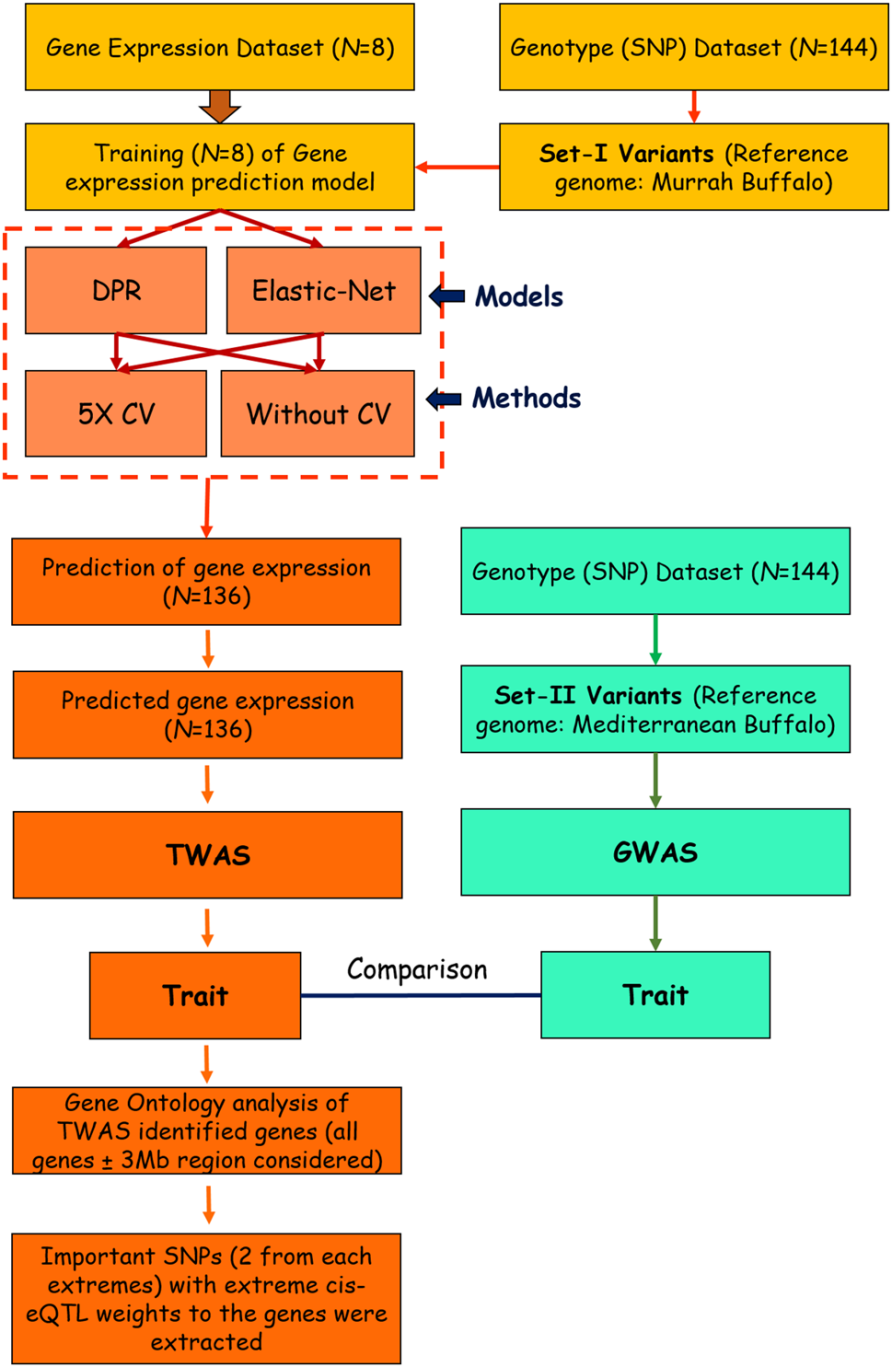
A brief workflow of the present study depicting steps from data acquisition to final genome-wide and transcriptome-wide associations.

For training and prediction of the $G\hat{R}eX$, both non-parametric Bayesian DPR method and parametric Elastic-Net model were used each with 5X cross validation (CV) and without any CV. Training, prediction, and association with phenotypes was accomplished using TIGAR: An Improved Bayesian Tool for Transcriptomic Data Imputation Enhances Gene Mapping of Complex Traits. ~ 15 simulations were run to test the appropriate parameters for the training model. Training was done with fivefold cross validation and without cross validation (leaving two out of 8 samples rotationally per iteration and training with all 8 samples) for both DPR and Elastic-Net model. An overall TWAS workflow is presented in Fig. 1.

Only the additive genetic effects of the cis-SNPs on genes were estimated as non-additive such as dominance and interaction effects tend to be overestimated in the small training samples, and estimation of only additive effects provide better prediction accuracy. SNPs were excluded if missing rate exceeded 0.2. Those SNPs having a MAF < 0.01 and deviating from the Hardy–Weinberg Equilibrium at p < 0.0001 were also excluded from the training. For training and individual level associations 1,64,830 QC passed Set-I SNPs were used. Association was performed based on the following model.

3 $${\text{f}}\left( {{\text{E}}\left[ {{\text{Y}}\left| {{\text{X}},{\text{C}}} \right.} \right]} \right) = \upeta {\text{C}} + \upbeta G\hat{R}eX$$

where, f(.) is a pre-specified link function, which is set as identity function for the quantitative phenotype, [Y|X,C]: Phenotype given genotype matrix *X*_test_ and covariate matrix C, H_0_: β = 0 in Eq. (3).

Same covariates as that of GWAS i.e., AFC, Birth weight, and 1st two components of MDS of set-II variants were taken to maintain homogeneity. TWAS was performed for 305 DMY, PY, WA, fat% and SNF% in the test individuals (*N* = 136). A detailed methodology is provided in the supplementary file for methods.

### Comparison of GWAS and TWAS results

Chromosome wise TWAS results for each trait and each model were combined to generate TWAS Manhattan plots. Manhattan plots generated from GWAS for each trait were compared with the TWAS Manhattan plots. Based on the significant genes and peak signals from the TWAS results via the DPR method, important genes were identified for the studied lactational traits. A TWAS hit gene’s midpoint position ± 1.5 Mb stretch was checked for presence of other potential TWAS hits those couldn’t be detected directly from the TWAS. Genes that are lying within that stretch of 3 Mb was considered for pathway enrichment. The pathways enriched with p < 5 × 10^–2^ were considered to be significantly enriched for the respective traits and the genes involved in those pathways were selected as probable candidate genes through the online gene ontology analysis platform gProfiler. Genes near the ± 20 kb of the GWAS hit SNPs and TWAS hit genes were compared for any shared genes among all methods. SNPs having highest positive and negative weights on prediction of those genes were recommended as important markers for further studies.

### Ethics statement

The animal study was reviewed and approved by the ICAR-National Dairy Research Institute (IAEC). All experiments were performed in accordance with relevant guidelines and regulations.

## Results

### Genotyping by sequencing and total mRNA sequencing

An average of 2.31 million each of forward and reverse reads of 151 base pairs (bp) were obtained per sample after the ddRAD sequencing. The average GC content of the reads was 51.20% with 35–40 Phred score (Q). Raw reads were aligned to the latest reference genome GCF_019923935.1_NDDB_SH_1_genomic.fna with 97.59% mapping rate and 52.34 average mapping quality. Clean BAM files obtained after sorting, trimming, and duplicate removal were used for variant calling and 57,92,182 polymorphic sites containing 55,60,412 SNPs and 2,31,770 indels were obtained. A total of 1,64,830 SNPs (Set-I) those passed QC were finally retained for GReX prediction for TWAS. For GWAS, variants (Set-II) were called using Mediterranean reference buffalo genome as the variants called using latest reference genome showed high genomic inflation when used for GWAS. The reads were mapped to the Mediterranean reference genome with 95.45% mapping rate and 32.73 average mapping quality. A total of 5,804,693 variants were obtained among which 5,544,733 were SNPs and 2,59,960 were indels. After applying quality control threshold for GWAS (Table S1), a total of 39,019 SNPs were retained for the GWAS.

An average of 30.68 GB of raw data per sample in paired end mRNA sequencing was obtained. The average genome coverage was 2.59X with 87.06% mapping rate to the reference genome (GCF_019923935.1_NDDB_SH_1). After quality control, number of reads counted as fragments for the paired end data and an average of 38.73% of reads were mapped to exonic region (Table 1). Total 33,347 features were counted as genes and their expression values were obtained.

**Table 1:** RNAseq feature count and assignment details obtained after counting the fragments via featureCounts v2.0.1.

Sample	Aligned to genes	Exonic	Intronic	Intergenic	Intronic/intergenic overlapping exon
T1	20901928	25.93	27.02	47.05	1.48
T2	7630593	14.97	37.16	47.87	0.74
T3	57004368	59.32	16.52	24.16	1.17
T4	1880851	6.43	64.39	29.19	0.4
T5	99543722	69.69	13.67	16.64	0.78
T6	66600631	63.7	15.76	20.54	1.66
T7	2286347	10.23	33.03	56.74	1.69
T8	20675305	59.6	33.22	7.19	19.24
Average	34565468	38.73	30.09	31.17	3.39

### Genome-wide association study (GWAS)

GWAS was performed for 1st lactation 305 days milk yield, peak yield, wet average, fat% and SNF% taking AFC, birth weight, and 1st two MDS components as covariates. The association results for each trait are depicted through Manhattan plot along with the association results of TWAS for that particular trait. The Q-Q plot signifying the distribution of p-values with respect to the test hypothesis is presented in Fig. S1. The top ten SNPs with respect to highest –logP values and the genes in their vicinity of ± 20 kb are presented in the Table 2.

**Table 2:** Genes annotated ± 20 kb of the top ten SNPs having lowest p-values in GWAS for each trait.

Traits	Chromosome	Position	Genes
Milk yield	2	18564231	*LGSN, CYFIP1*
	3	152863609	*TBX19, FAM78B*
	3	88029567	*TBX19, FAM78B*
	10	19195382	*KCNN2, CELF6*
	11	30281597	*ZC3H8, MTLN, NPHP1*
	16	47885286	*TMEM183A*
	16	74362826	*PLXNA2*
	17	29702216	*ABHD18*
	20	13906580	*PANK3, SGTB, TRAPPC13, TRIM23*
	24	36865371	*METTL4, NDC80*
Wet average	3	88029567	*TBX19, FAM78B*
	4	44518782	*TMBIM7, PMPCB, DNAJC2*
	6	112228435	*APELA, LARP7*
	6	112228371	*APELA, LARP7*
	10	48419016	*MCC, KCNN2*
	10	16657423	*MCC, KCNN2*
	11	30281597	*ZC3H8, MTLN, NPHP1*
	18	37277083	*UQCRFS1, SF3B3*
	21	39649505	*NDN*
	24	28499463	*PARD6G*
Peak yield	2	18564231	*LGSN, CYFIP1*
	3	152863609	*TBX19, FAM78B*
	4	44518782	*TMBIM7, PMPCB, DNAJC2*
	10	16657423	*MCC, KCNN2*
	10	17324461	*UACA*
	11	30281597	*ZC3H8, MTLN, NPHP1*
	11	24583445	*ZC3H8, MTLN, NPHP1*
	20	13906580	*PANK3, SGTB, TRAPPC13, TRIM23*
	23	32648766	*PRIM2*
	24	28499463	*PARD6G*
Fat%	3	162275901	*TBX19, FAM78B*
	3	91898446	*FAM78B, SSBP3*
	4	48399143	*VSTM2A, TMBIM7*
	4	40204848	*VSTM2A,TMBIM7*
	5	84739212	*KRAS, ETFRF1*
	8	58357861	*MFSD14B*
	16	66495537	*TMEM183A*
	17	15895247	*KLHL2*
	20	34215807	*SPZ1,PANK3*
	22	15166346	*MRPS24*
SNF%	3	47608245	*TBX19, FAM78B*
	3	66715572	*FAM78B, MIR2285BB DNAJB4, FUBP1*
	9	96723520	*PTP4A1*
	10	24182429	*MCC, KCNN2*
	11	35249431	*ZC3H8, MTLN, NPHP1*
	13	12532412	*PLCB1, ECHDC3*
	14	54067539	*C14H8orf33, ZNF34, ARHGAP39*
	16	10198062	*TMEM183A*
	20	45882062	*SPZ1, PANK3*
	23	41123236	*PRIM2*

### Transcriptome-wide association study (TWAS)

#### Model training and gene-expression prediction

Set-I SNPs (1,64,830) were used to predict 26,956 genes which were further used to perform TWAS. Training accuracy was higher in case of ENET model through both with- and without cross-validation with an average of 0.67 while through DPR model training accuracy was 0.49 without cross-validation and 0.48 with cross-validation. λ value of was found to be 0.32 on an average across the chromosomes in the ENET model for both the methods indicating that regression coefficients were moderately shrinked with α when assumed to be 0.5 and with α determined by cross-validation. Chromosome wise training accuracy across all models and methods is presented in Table 3. The cis-QTL weights for the SNPs predicted through the DPR model with and without cross-validation were same; hence, the prediction of gene expression levels and their association with the respective traits was done only for the DPR model without cross-validation method. While separate association results were obtained for ENET with and without cross-validation methods.

**Table 3:** Chromosome-wise DPR and ENET model performances in gene-expression training and gene prediction.

Models	Training R^2^				λ		Total number of genes used for training	No. of genes imputed for expression value			
	DPR		ENET		ENET			DPR		ENET	
Chromosome no	Without_CV	With_CV	Without_CV	With_CV	Without_CV	With_CV		Without_CV	With_CV	Without_CV	With_CV
1	0.4931	0.4898	0.6482	0.6375	0.3471	0.3584	1781	1446	1325	1168	1120
2	0.4823	0.4756	0.6514	0.6451	0.3424	0.3476	2504	2007	1798	1650	1598
3	0.4814	0.478	0.7142	0.7068	0.2797	0.2867	2854	2306	2044	2057	1986
4	0.493	0.4905	0.6735	0.6684	0.3125	0.3181	2160	1725	1568	1488	1444
5	0.454	0.4514	0.7033	0.694	0.2839	0.2929	1856	1527	1385	1332	1284
6	0.5022	0.5028	0.6778	0.675	0.312	0.3172	1910	1574	1428	1316	1282
7	0.5093	0.5025	0.6563	0.6518	0.3341	0.3368	897	749	693	601	582
8	0.4697	0.4627	0.6316	0.625	0.3586	0.3656	1242	1008	927	797	775
9	0.4679	0.4695	0.7196	0.709	0.2737	0.2843	1792	1434	1285	1303	1248
10	0.4712	0.469	0.6356	0.6239	0.3558	0.3682	808	645	587	524	500
11	0.5042	0.504	0.6584	0.6499	0.3292	0.3365	1556	1158	1024	1041	1001
12	0.498	0.4926	0.6901	0.6842	0.2931	0.2996	1410	1140	996	976	938
13	0.4779	0.4778	0.6587	0.6423	0.3304	0.3475	633	478	427	415	391
14	0.4787	0.4932	0.7128	0.7037	0.2802	0.2873	1191	959	856	861	824
15	0.4924	0.4936	0.6688	0.6575	0.3227	0.334	785	652	579	531	503
16	0.4728	0.4678	0.6155	0.608	0.3768	0.3843	1390	1131	1023	870	835
17	0.502	0.5035	0.6696	0.6649	0.3163	0.3201	903	770	673	620	604
18	0.5128	0.5101	0.7685	0.7626	0.2284	0.2339	1722	1401	1241	1341	1296
19	0.4824	0.4789	0.6199	0.6151	0.3587	0.3651	526	421	392	333	321
20	0.4771	0.4701	0.6435	0.6428	0.3356	0.3373	913	736	661	613	595
21	0.4863	0.4783	0.7197	0.7153	0.2691	0.2735	796	651	584	584	568
22	0.5001	0.4985	0.6838	0.683	0.3129	0.3151	547	440	404	375	366
23	0.4848	0.4903	0.6773	0.6702	0.2985	0.305	650	542	493	451	434
24	0.4948	0.493	0.7448	0.7463	0.2444	0.2423	998	866	782	753	728
X	0.4793	0.4807	0.5828	0.5762	0.3916	0.3984	1486	1190	1112	904	871
Average	0.49	0.48	0.67	0.67	0.32	0.32	**–**	**–**	**–**	**–**	**–**

#### Transcriptome-wide association results

**Figure 2:**
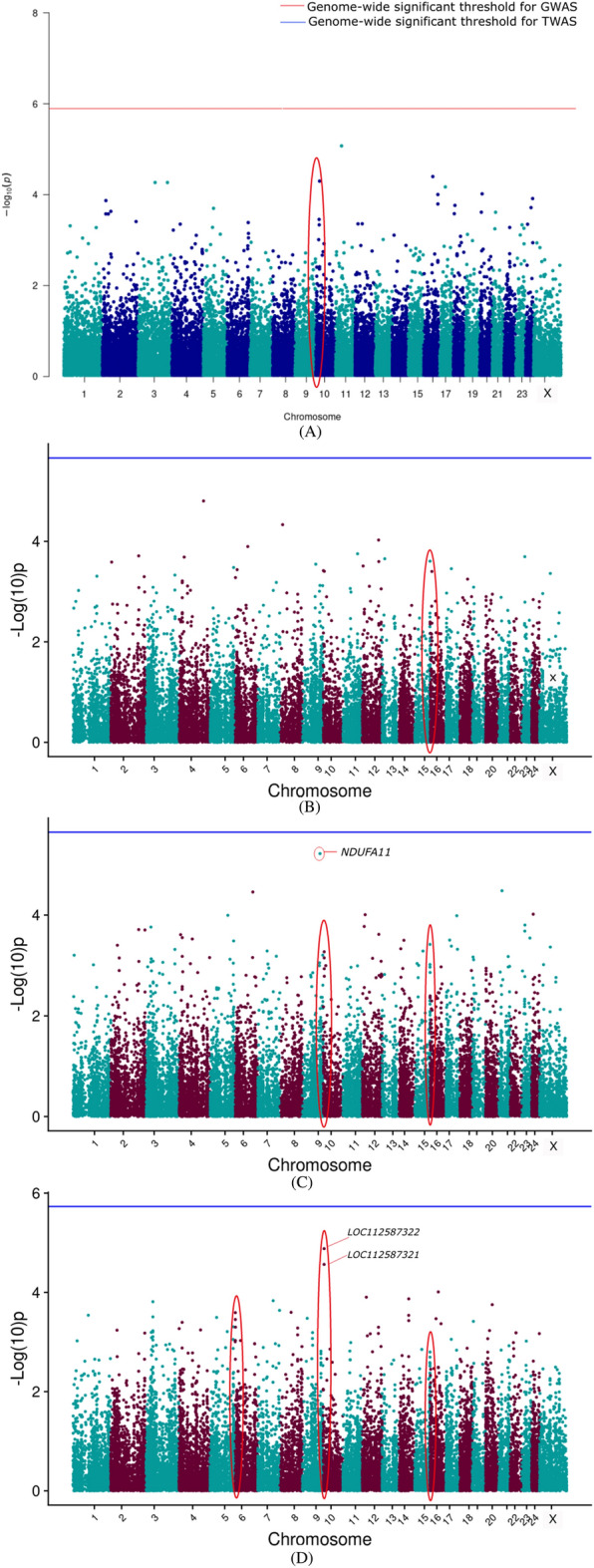
Manhattan plot showing the results of associations with the 305 DMY (**A**) GWAS (**B**) TWAS by ENET without CV (**C**) TWAS by ENET with 5X CV, and (**D**) TWAS by DPR without CV models. The red line indicates genome-wide *p-*value threshold (expressed as –log_10_*P*) corresponding to Bonferroni corrected *p-*values, above which the SNPs are considered to be significantly associated with the trait in GWAS, while the blue line indicates genome-wide significant threshold of Bonferroni corrected *p-*values for TWAS models. ^†^The red ovals surrounding various genomic region suggest the significant SNP/genes and peak association signals.

**Figure 3:**
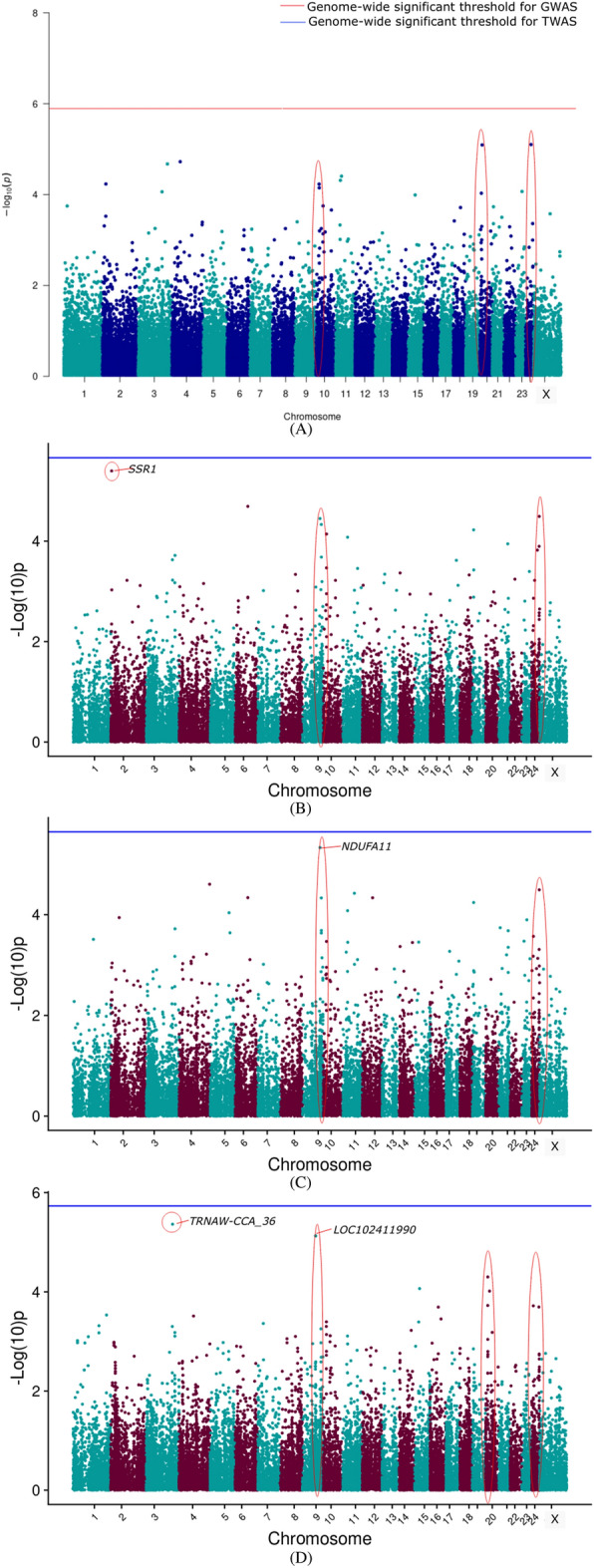
Manhattan plot showing the results of associations with the PY (**A**) GWAS (**B**) TWAS by ENET without CV (**C**) TWAS by ENET with 5X CV, and (**D**) TWAS by DPR without CV models. The red line indicates genome-wide *p-*value threshold (expressed as –log_10_*P*) corresponding to Bonferroni corrected *p-*values, above which the SNPs are considered to be significantly associated with the trait in GWAS, while the blue line indicates genome-wide significant threshold of Bonferroni corrected *p-*values for TWAS models. ^†^The red ovals surrounding various genomic region suggest the significant SNP/genes and peak association signals.

**Figure 4:**
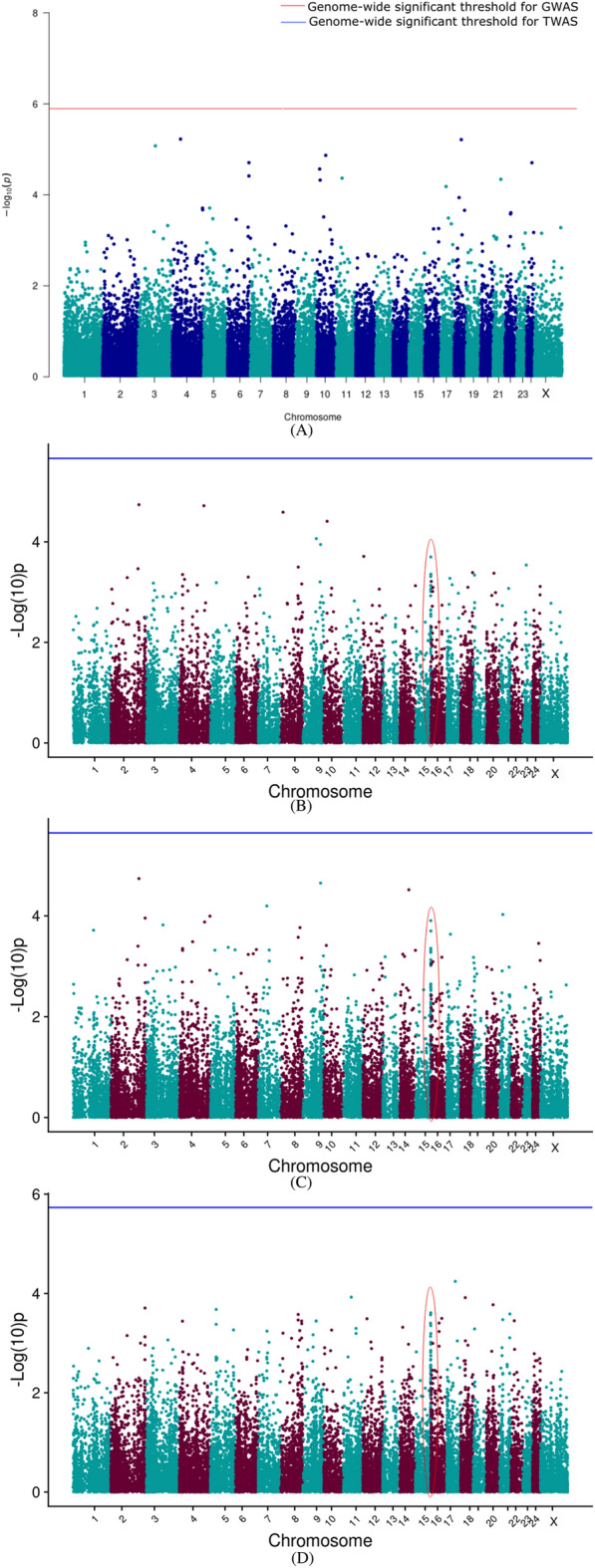
Manhattan plot showing the results of associations with the WA (**A**) GWAS (**B**) TWAS by ENET without CV (**C**) TWAS by ENET with 5X CV, and (**D**) TWAS by DPR without CV models. The red line indicates genome-wide *p-*value threshold (expressed as –log_10_*P*) corresponding to Bonferroni corrected *p-*values, above which the SNPs are considered to be significantly associated with the trait in GWAS, while the blue line indicates genome-wide significant threshold of Bonferroni corrected *p-*values for TWAS models. ^†^The red ovals surrounding various genomic region suggest the significant SNP/genes and peak association signals.

**Figure 5:**
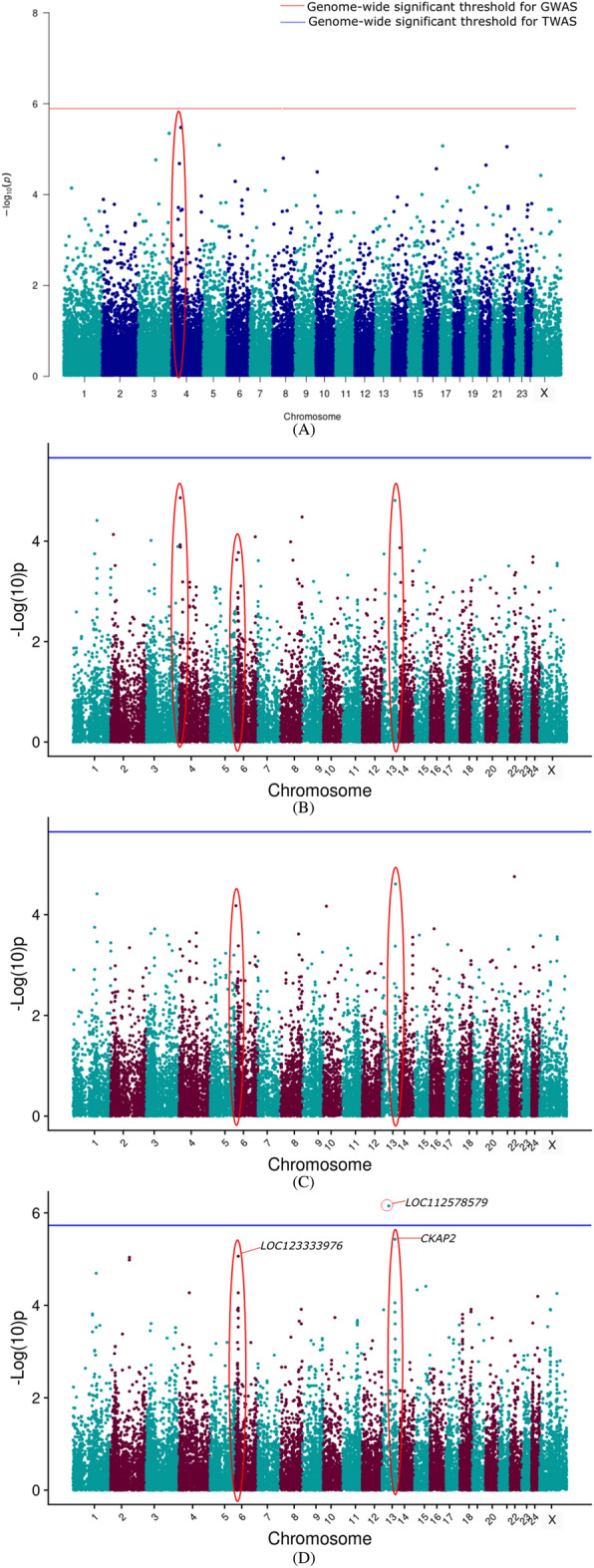
Manhattan plot showing the results of associations with the Fat% (**A**) GWAS (**B**) TWAS by ENET without CV (**C**) TWAS by ENET with 5X CV, and (**D**) TWAS by DPR without CV models. The red line indicates genome-wide *p-*value threshold (expressed as –log_10_*P*) corresponding to Bonferroni corrected *p-*values, above which the SNPs are considered to be significantly associated with the trait in GWAS, while the blue line indicates genome-wide significant threshold of Bonferroni corrected *p-*values for TWAS models. ^†^The red ovals surrounding various genomic region suggest the significant SNP/genes and peak association signals.

**Figure 6:**
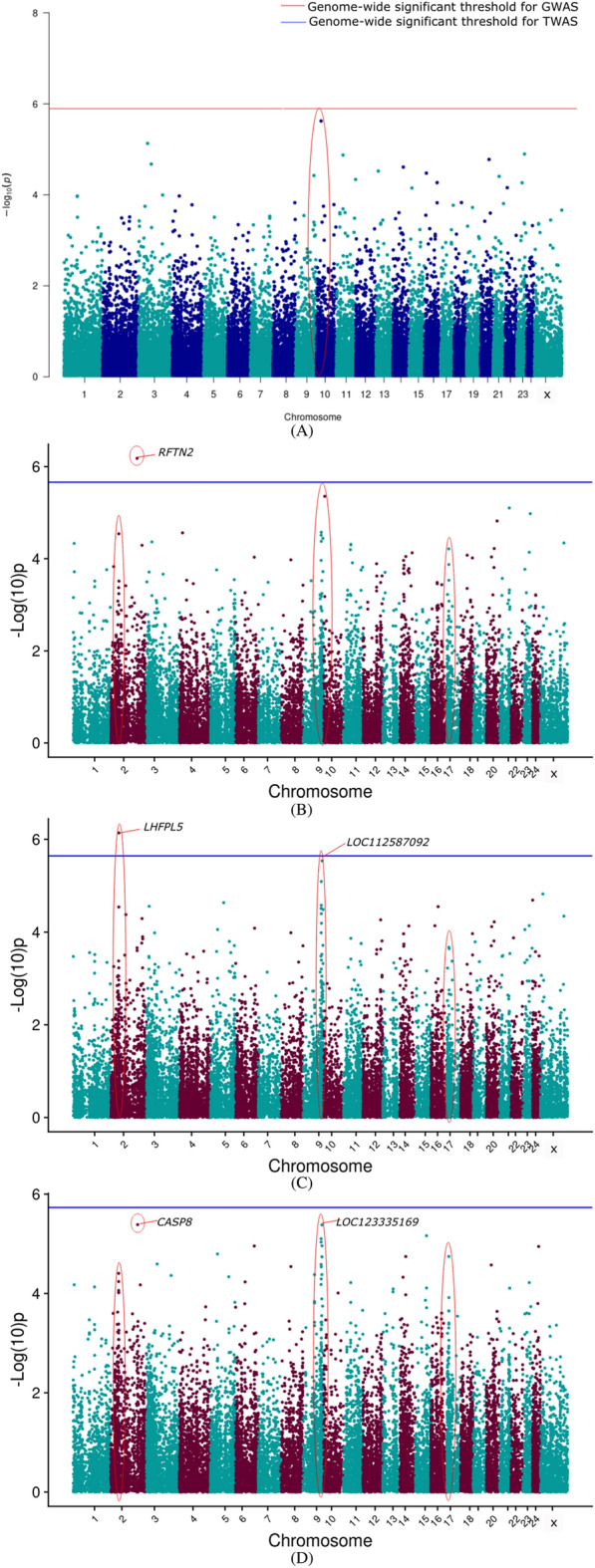
Manhattan plot showing the results of associations with the SNF% (**A**) GWAS (**B**) TWAS by ENET without CV (**C**) TWAS by ENET with 5X CV, and (**D**) TWAS by DPR without CV models. The red line indicates genome-wide *p-*value threshold (expressed as –log_10_*P*) corresponding to Bonferroni corrected *p-*values, above which the SNPs are considered to be significantly associated with the trait in GWAS, while the blue line indicates genome-wide significant threshold of Bonferroni corrected *p-*values for TWAS models. ^†^The red ovals surrounding various genomic region suggest the significant SNP/genes and peak association signals.

The TWAS results are presented as a Manhattan plot for the different models along with GWAS results in Figs. 2, 3, 4, 5, 6 for 305 days milk yield, peak yield, wet average, fat%, and SNF%, respectively. The top 10 genes having the lowest *P*-value along with their FDR corrected *P*-values were identified and are given in the supplementary document (Tables S2–S6).

TWAS results for 305 days milk yield, peak yield, and wet average were checked to assess the role of important genes associated with milk production. From the corresponding Manhattan plots of TWAS for 305 days milk yield, peak signals of probable associations were observed on BBU10 at ~ 4 Mbp, BBU15 at ~ 37 Mbp and ~ 81 Mbp, and BBU6 at ~ 1 Mbp. Notably, the well-known candidate gene *DGAT1* for milk yield is positioned at 81362831–81371652 bp on BBU15. Similar peak patterns were observed on the BBU15 at ~ 81 Mbp from the TWAS results for wet average. Apart from the TWAS results of the DPR model, TWAS for 305 DMY through Elastic Net model with 5X cross-validation revealed *NDUFA11* on BBU9 at ~ 92 Mbp to be the top gene with lowest p-value. *NDUFA11* was also found as the top gene with lowest p-value for the peak yield TWAS through Elastic Net model with cross-validation. From the peak yield TWAS through DPR model, peak signals were observed at BBU2 at ~ 17 Mbp, BBU20 at ~ 11 Mbp, BBU24 at ~ 38Mbp, and BBU9 at ~ 69Mbp. Though no significant associations were observed for milk production traits through TWAS, FDR corrected *P*-values in DPR model denoted significant association of *TRNAW-CCA_36, LOC102411990, LOC102415512, LOC102416173*, and *LOC123335276* with the peak yield. *LHFPL5* and *RFTN2* were significantly associated with the SNF% in the ENET models.

For milk production, *CREB3L3, GNA15, GNG7, MAP2K2, MAP2K7, SHC2*, and *ADCY9* were found to be involved in the Relaxin signaling pathway. *TNFRSF11B, AMH, CCL25, CD70, EBI3, IL13, IL4, IL5, TNFSF14, TNFSF9, GDF9, LTA, LTB, TNF,* and *TNFRSF12A* were significantly enriched in the cytokine-cytokine receptor interaction. *IL13, IL4, IL5, MAP2K2, MAP2K7, VAV1,* and *TNF* were significantly enriched in the Fc epsilon RI signaling (FcεRI) pathway. *GNA11, MAP2K2, HCN2, KISS1R*, and *GABBR1* were enriched in the GnRH secretion pathway. *CREB3L3, GNA11, MAP2K2, SHC2*, and *ADCY9* were enriched in the Growth hormone synthesis, secretion and action pathway. A novel uncharacterized gene *LOC112578579* at BBU13 was found to be significantly associated with Fat% in the DPR without CV TWAS model. Along with that, peak signals were found on BBU13 at ~ 68 Mbp, and BBU6 at ~ 16 Mbp. For Fat%, AMPK signaling pathway was significantly highlighted by both KEGG and Wiki pathways. The genes found to be involved in the AMPK signaling were *CCNA1, CAB39L, CREB3L4, CRTC2* and *FOXO1*. *FOXO1* was also significantly enriched in the constitutive androstane receptor pathway and adipogenesis. *EBPL* gene was found to be significantly enriched for the biological process sterol metabolism.

Though DPR was considered as the most suitable model for our population, genes such as *LHFPL5* and *RFTN2* were found to be significantly associated with SNF% in the ENET 5X CV and ENET without CV models, respectively. Meanwhile, in the TWAS results of the DPR model, peak signals were observed on BBU2 at ~ 141.9 Mbp, BBU9 at ~ 100 Mbp, and on BBU17 at ~ 10 Mbp. *AOX1, IL27RA, TYK2*, and *PTPN11* genes were significantly enriched in the JAK-STAT signaling pathway. Genes such as *SDS* and *SDSL* were enriched for the valine, leucine and isoleucine biosynthesis and cysteine and methionine metabolism pathway.

The novel genes identified for milk production, Fat%, and SNF% recommended for further studies are given in the Tables 4, 5, 6. The genes identified through the TWAS were then further checked for the SNPs having highest negative and positive weights for the prediction of that particular gene. The two top SNPs having the most negative and positive effect sizes for each TWAS highlighted genes were filtered from the gene prediction result files to obtain the important SNPs to be used as important markers in future selection programs (Tables S7–S9).

**Table 4:** Chromosomal position and description of candidate genes identified for milk production.

Chr^a^	Start	End	Gene	Description
24	38853457	38939708	*ADCY9*	Adenylate cyclase 9
9	91338776	91349987	*CREB3L3*	cAMP responsive element binding protein 3 like 3
15	81362831	81371652	*DGAT1*	Diacylglycerol O-acyltransferase 1
15	25908087	25956298	*EIF3E*	Eukaryotic translation initiation factor 3 subunit E
9	69978056	69979015	*LOC102411990*	Olfactory receptor 5F1-like
15	81381535	81387066	*LOC112579064*	WAS/WASL-interacting protein family member 2-like
17	44465782	44577887	*LOC112579963*	Uncharacterized
2	17707146	17707254	*LOC112583021*	U6 spliceosomal RNA
10	4649442	4651633	*LOC112587322*	Uncharacterized
9	91286096	91309185	*MAP2K2*	Mitogen-activated protein kinase kinase 2
9	94502825	94512982	*MAP2K7*	Mitogen-activated protein kinase kinase 7
6	1079791	1160577	*MPZL1*	Myelin protein zero like 1
24	38138282	38147034	*NAGPA*	N-acetylglucosamin × 10-1-phosphodiester alpha-N-acetylglucosaminidase
9	92721700	92726796	*NDUFA11*	NADH:ubiquinone oxidoreductase subunit A11
20	11406835	11508065	*PPP4R4*	Protein phosphatase 4 regulatory subunit 4
3	140320581	140320652	*TRNAW-CCA_36*	Transfer RNA tryptophan (anticodon CCA)

^a^Denotes *Chromosome no.*

**Table 5:** Chromosomal position and description of candidate genes identified for milk fat percentage.

Chr^a^	Start	End	Gene	Description
13	70503653	70589374	*CAB39L*	Calcium binding protein 39 like
13	64545661	64557188	*CCNA1*	Cyclin A1
13	68036475	68062355	*CKAP2*	Cytoskeleton associated protein 2
6	16516950	16522814	*CREB3L4*	cAMP responsive element binding protein 3 like 4
6	16530385	16540109	*CRTC2*	CREB regulated transcription coactivator 2
13	70284989	70380518	*EBPL*	EBP like
13	67652157	67742085	*FOXO1*	Forkhead box O1
13	35434571	35434674	*LOC112578579*	U6 spliceosomal RNA
6	16551688	16560448	*LOC123333976*	Uncharacterized

^a^Denotes *Chromosome no.*

**Table 6:** Chromosomal position and description of candidate genes identified for milk SNF percentage.

Chr^a^	Start	End	Gene	Description
2	141239521	141309458	*AOX1*	Aldehyde oxidase 1
2	141982022	142006184	*CASP8*	Caspase 8
17	10125393	10180481	*GCN1*	GCN1activator of EIF2AK4
9	99418351	99442312	*IL27RA*	Interleukin 27 receptor subunit alpha
9	100515335	100516339	*LOC123335169*	Olfactory receptor 7A10-like
17	10848425	10919255	*PTPN11*	Protein tyrosine phosphatase non-receptor type 11
2	41189571	41219947	*RAB44*	RAB44, member RAS oncogene family
17	11609354	11617523	*SDS*	Serine dehydratase
17	11628968	11642021	*SDSL*	Serine dehydratase like
9	96284490	96312587	*TYK2*	Tyrosine kinase 2

^a^Denotes *Chromosome no.*

**Figure 7:**
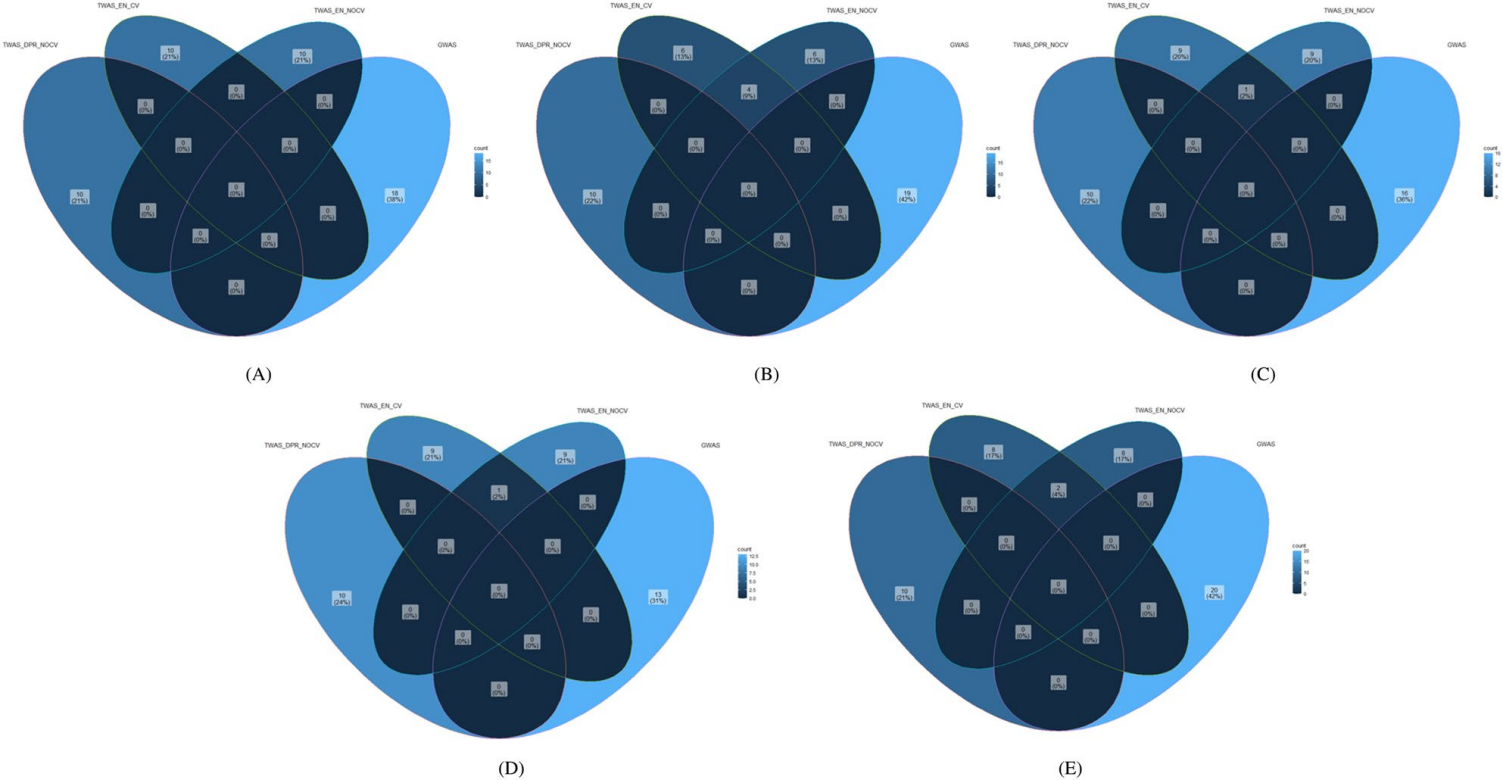
Venn diagram showing number of shared genes between the top ten list of GWAS and TWAS. (**A**) 305 days milk yield, (**B**) Peak yield, (**C**) Wet average (**D**) Fat %, and (**E**) SNF % (DPR without CV, ENET with CV, and ENET without CV) (the darkest blue colour indicates no shared genes among the methods).

The genes identified through GWAS and TWAS hits were checked for common genes between them but no such common genes could be found (Fig. 7). Only ENET CV and without CV method showed 1, 4, and 2 genes for fat%, peak yield, and SNF%, respectively.

## Discussion

This study is a first of its kind to be conducted in dairy animals to obtain transcriptome-wide association of predicted gene expression levels with economically important production traits of Murrah buffalo. Since the conception of TWAS as a post-GWAS prioritization tool in humans to identify and delineate the biological function of causal genes responsible for various diseases and quantitative phenotype viz. height, the field of animal science is lagging way behind in exploring the same in livestock species to augment genomic selection programs. Though GWAS have been very effective and one of the most widely used method to map several causal loci for complex traits; yet the biological gaps are still evident. The majority of GWAS-hit loci lie in non-coding regions and, even though they might play a role in gene expression regulation, its physiological perspective is unclear. In dairy animals, most of the variants contributing to complex lactation traits have not yet been identified, as their effect sizes are too small to be detected at current GWAS sample sizes. Hence, the need to perform TWAS like post-GWAS studies in animal species couldn’t be undermined any longer and the present study is an application of the concept in one of the major dairy animal species of India i.e., Murrah buffalo.

Genotyping by sequencing (GBS) using double digestion RAD tag technology have already been proven to be efficacious in terms of cost, speed of genotyping, robustness and is generalized to any species^32,33^. GBS aids in obtaining a large number of genome-wide SNP information for exploring within-species diversity, constructing haplotype maps and performing genome-wide association studies (GWAS)^34^, at a lesser expense than other contemporary methods. The ddRAD genotype information generated in the present study provided 1X genome coverage, and upon alignment to the reference genome 97.59% mapping rate was observed proving its suitability to be used in the GWAS and TWAS studies in the Murrah buffalo.

The transcriptome information for the present study was obtained by sequencing total mRNAs from mammary epithelial cells of lactating animals. Milk is a heterogeneous source of somatic cells composed of lymphocytes, neutrophils, macrophages and exfoliated epithelial cells^35^. The expression of genes involved in cell turnover, milk synthesis, or hormonal regulation in the mammary tissue is a key determining factor for milk production in ruminants. The applicability of the milk-isolated MECs to analyze mammary gene expression has been substantiated through many studies, as the gene transcript variations were also in accordance with milk yield and composition variations. Mammary epithelial cells (MEC) are unique in the way they are involved in the synthesis and secretion of milk, despite popularity of milk somatic cells in analysis of the gene expression for milk synthesis in ruminants^36^. Milk isolated mammary epithelial cells produce similar transcript variation profile that is consistent with variations in milk yield and compositions^37^. The genome-wide gene expression levels obtained from the MECs are well representation of the genome-wide lactation specific genes in the present study.

As in one of our previous studies, we highlighted the reasons for taking a GWAS sample size in hundreds as optimum to find significant results considering the buffalo farming scenario in Indian sub-continent^23^. Majority of organized buffalo farms in India have a herd size of 250–500 and only 100–200 breedable population, along with an absence of any functional buffalo sequencing consortium in India, renders sequencing of few hundreds of animals with sufficient genetic diversity to be feasible. In a such a case, when we considered genotyping 144 unrelated Murrah buffaloes, proportionately, for performing TWAS, total mRNA sequencing of 8 individuals were considered optimum, and the results obtained through TWAS evidently showed the robustness of the method and applicability in Murrah population despite the low sample size.

The gene expression prediction models i.e., Elastic-Net and DPR with 8 samples were trained. Nagpal et al.^14^ had discussed the higher power of TWAS using DPR over TWAS using Elastic-Net model. They have shown through a series of simulation along with real data study with ROS/MAP data that indicated the superior performance of DPR model over Elastic-Net model implemented in the PrediXcan, in terms of TWAS power and imputation R^2^. The advantage of DPR model lies in its flexibility of non-parametric Bayesian modelling that predicts higher number of genes with a better imputation R^2^ at causal gene proportions $\ge$ 0.01 and at h_e_^2^ $\le$ 0.2. As, the lactation traits are of polygenic nature and are controlled by many genes, proportions of causal cis-SNPs are expected to be much higher and there is no prior information regarding the effect size distribution of cis-eQTLs, the DPR method of gene expression prediction can be considered as a choice of model for such a case. This assumption was also consistent with the results obtained in the present study that showed higher number of predicted genes through both DPR 5X cross-validation and without cross-validation methods than Elastic-Net model. DPR without CV model predicted 12.16% more number of genes than ENET without CV model, while DPR 5X CV model predicted 6.59% higher genes than ENET 5X CV. DPR without CV model predicted 80.92% of the genes used for training. The average training R^2^ was ~ 0.48 in DPR model while it had significantly higher value of ~ 0.67 through ENET model. Higher training R^2^ than 0.5 may seem as a model overfitting due to small sample sizes, which was also observed by Parrish et al.^38^ in their TWAS study with 49 tissue types. As DPR model is reported to be generalized, flexible, robust, and accounts for better prediction performance across broad genetic architectures^38,39^, and also in the present study it predicted higher number of genes with a reliable training R^2^, we selected DPR as choice of gene expression prediction model for our Murrah population. However, the weights predicted for the DPR model through 5X CV and without CV were found to be same for both which may be due to the small sample size. Hence, only the DPR without CV model was included for further study along with both ENET models.

A final association study was conducted for different 1^st^ lactation traits with gene expressions predicted using various models and the results were compared with GWAS results. For comparison purpose the phenotype association model was same for both the GWAS and TWAS. The TWAS results from DPR without CV, ENET 5X CV, and ENET without CV showed higher numbers of true positives than GWAS for all the traits. The *P*-values of associations were adjusted for multiple testing by Bonferroni’s correction and FDR-BH (False Discovery Rate by^27^. Significant associations were observed after adjusting for multiple testing for fat% and SNF% using TWAS while no such associations could be observed in GWAS. The FDR of the top 10 genes for different traits in various TWAS models shows the number of suggestive associations that could be truly positive but couldn’t pass the Bonferroni’s threshold possibly due to the small sample size.

The present study revealed Relaxin-signaling pathway as a regulator for milk yield. Previously, *ADCY5* of the Adenylate Cyclase family was reported to be a candidate gene for regulating milk yield in buffaloes^40^, which also signifies the role of Adenylate Cyclase family genes in regulation of milk yield. Upon the network analysis, it was observed that *ADCY5* is also a key regulator of the Relaxin-signaling pathway. Although, Relaxin is a well-known hormone secreted during pregnancy in some species to soften the cervix and prepare the reproductive tract for parturition, it was also reported to have major mammogenic role in sows^41^. *CREB3L3* is also reported to act as a central regulator of energy homeostasis through AMP signaling pathway in dairy cows^42^. Ye et al.^40^ reported *INHBA* and *INHBB* to be involved in cytokine-cytokine interaction pathway and this pathway has been reported to be a significant regulator of milk yield. Ahlawat et al.^43^ reported that genes down-regulated in milk somatic cells of buffaloes were significantly enriched in cytokine receptor interaction pathway. Several other genes of cytokine receptor families were identified to be involved in heat tolerance in water buffaloes^44^.

In Nili-Ravi buffaloes, Prolactin (*PRL*) a major gene involved in mamogenesis, regulation of milk protein, and milk secretion is reported to be regulated by the cytokine-cytokine interaction pathway^45^. Ye et al.^40^ reported *INHBA* and *INHBB* as candidate genes in regulation of milk yield that were also significantly enriched in the cytokine-cytokine receptor interaction pathway in the present study. Several genes from the TWAS results along with previously reported *PIK3R1*^40^ were significantly enriched in the Fc epsilon RI signaling (FcεRI) pathway. *FcεRI* is required for cell membrane expression and intracellular signal transduction. Milk production TWAS genes also found to be involved in GnRH secretion and growth hormone synthesis, secretion and action pathway. Several reports indicate that growth hormone and growth hormone receptor genes play a vital role in growth of mammary gland in lactating females and regulation of milk yield. The *GHR* gene is implicated in lipid and carbohydrate metabolism and maintaining lactation^46^. *GHR* polymorphism has been reported to be associated with milk yield in buffaloes^47^.

AMPK signaling pathway is previously reported to be involved in regulation of milk production^48^, milk fat and protein synthesis^49^. The AMP-activated protein kinase (*AMPK*) was also reported to control lipid and lactose synthesis in bovine mammary epithelial cells^50^. AMPK signaling pathway was also reported to be involved in modulation of milk yield in buffaloes with *ELAVL1, RAB11B, ADIPOR2, ADRA1A, INSR, LEP, PIK3CA, SCD*, and *TSC1* genes as nodes^51^. *FOXO1* is a member of fork-head family of transcription factors that plays a vital role in gluconeogenesis in the liver^52^. Jacometo et al.^53^ also suggested the role of *FOXO1* in milk fat synthesis. *FOXO1* was reported to be differentially expressed for milk fat traits in Chinese Holstein cattle^54^. Sterol metabolism reported to be a critical regulator of milk fat synthesis in dairy cows and several sterol regulatory element-binding proteins have been characterized as the candidate genes for the milk fat synthesis in mammary epithelial cells of dairy cows^55^.

Many studies have highlighted the role of JAK-STAT signaling pathway in mammary gland development and milk production. Khan et al.^56^ have reviewed several works highlighting the role of this pathway in milk casein gene regulation and interaction of lactogenic hormone receptors with JAK-STAT pathway to regulate milk proteins. Prolactin receptor is also reported to regulate few JAK-STAT-associated proteins that balances the growth hormone in relation to milk protein yield^57^. Ji et al.^58^ have also highlighted the role of *STATs* in regulating the 5′ flanking regions of Whey acidic protein (WAP) that is expressed in the mammary gland.

Methionine is the limiting amino acid for the ruminants and is essential for the milk protein synthesis whereas, valine, leucine and isoleucine are also essential amino acids that are reported to be potentially limiting for milk protein synthesis^59^. As the genes identified in the study play important role in pathways regulating milk yield either directly or indirectly, they can be considered as candidate genes for milk yield and its composition traits.

## Conclusion:

In a dairy breeding program, the prior knowledge about the distribution of eQTL effect size is often not considered. Non-parametric Bayesian based method could be an excellent choice to predict the eQTL effects. DPR is a one such model of gene expression prediction, this model can accommodate across tissue information, which improves the prediction accuracy. Our study concludes that the TWAS in the Murrah buffaloes for lactation traits proved to be more robust and efficacious than conventional GWAS even when the sample size are not large. Reasons could be a higher statistical power associated with TWAS. We were able to map important causal genes and many true positive associations for almost all the traits even with a small sample size using TWAS approach.

### Supplementary Information

Supplementary Information 1.

Supplementary Information 2.

## Data Availability

The datasets generated during and/or analysed during the current study are deposited in the European Variation Archive repository, accession number PRJEB47270 (https://wwwdev.ebi.ac.uk/eva/?eva-study=PRJEB47270).

## References

[1] Cao C, Ding B, Li Q, Kwok D, Wu J, Long Q (2021). Power analysis of transcriptome-wide association study: Implications for practical protocol choice. PLoS Genet..

[2] De Camargo GMF, Aspilcueta-Borquis RR, Fortes MRS, Porto-Neto R, Cardoso DF, Santos DJA, Lehnert SA, Reverter A, Moore SS, Tonhati H (2015). Prospecting major genes in dairy buffaloes. BMC Genomics.

[3] El-Halawany N, Abdel-Shafy H, Abd-El-Monsif AS, Abdel-Latif MA, Al-Tohamy AF, Abd El-Moneim OM (2017). Genome-wide association study for milk production in Egyptian buffalo. Livest. Sci..

[4] Liu JJ, Liang AX, Campanile G, Plastow G, Zhang C, Wang Z, Salzano A, Gasparrini B, Cassandro M, Yang LG (2018). Genome-wide association studies to identify quantitative trait loci affecting milk production traits in water buffalo. J. Dairy Sci..

[5] Deng T, Liang A, Liang S, Ma X, Lu X, Duan A, Pang C, Hua G, Liu S, Campanile G, Salzano A (2019). Integrative analysis of transcriptome and GWAS data to identify the hub genes associated with milk yield trait in buffalo. Front. Genet..

[6] Abdalla M, Abdalla M (2022). A general framework for predicting the transcriptomic consequences of non-coding variation and small molecules. PLoS Comput. Biol..

[7] Gusev A, Ko A, Shi H, Bhatia G, Chung W, Penninx BW, Jansen R, De Geus EJ, Boomsma DI, Wright FA, Sullivan PF (2016). Integrative approaches for large-scale transcriptome-wide association studies. Nat. Genet..

[8] Zhu D, Yao S, Wu H, Ke X, Zhou X, Geng S, Dong S, Chen H, Yang T, Cheng Y, Guo Y (2022). A transcriptome-wide association study identifies novel susceptibility genes for psoriasis. Hum. Mol. Genet..

[9] Li X, Su X, Liu J, Li H, Li M, Li W, Luo XJ (2021). Transcriptome-wide association study identifies new susceptibility genes and pathways for depression. Transl. Psychiatry.

[10] Rowland B, Venkatesh S, Tardaguila M, Wen J, Rosen JD, Tapia AL, Sun Q, Graff M, Vuckovic D, Lettre G, Sankaran VG (2022). Transcriptome-wide association study in UK Biobank Europeans identifies associations with blood cell traits. Hum. Mol. Genet..

[11] Sun Y, Zhu J, Zhou D, Canchi S, Wu C, Cox NJ, Rissman RA, Gamazon ER, Wu L (2021). A transcriptome-wide association study of Alzheimer’s disease using prediction models of relevant tissues identifies novel candidate susceptibility genes. Genome Med..

[12] Kremling KA, Diepenbrock CH, Gore MA, Buckler ES, Bandillo NB (2019). Transcriptome-wide association supplements genome-wide association in Zea mays. G3 Genes Genomes Genet..

[13] Ropka-Molik K, Bereta A, Żukowski K, Tyra M, Piórkowska K, Żak G, Oczkowicz M (2018). Screening for candidate genes related with histological microstructure, meat quality and carcass characteristic in pig based on RNA-seq data. Asian-Austral. J. Anim. Sci..

[14] Nagpal S, Meng X, Epstein MP, Tsoi LC, Patrick M, Gibson G, De Jager PL, Bennett DA, Wingo AP, Wingo TS, Yang J (2019). TIGAR: An improved Bayesian tool for transcriptomic data imputation enhances gene mapping of complex traits. Am. J. Hum. Genet..

[15] Veturi, Y. & Ritchie, M. D. How powerful are summary-based methods for identifying expression-trait associations under different genetic architectures?. In *Pacific Symposium on Biocomputing 2018: Proceedings of the Pacific Symposium*, 228–239 (2018). PMC5785784 29218884

[16] Sambrook J, Russell DW (2006). Purification of nucleic acids by extraction with phenol: chloroform. Cold Spring Harb. Protocols.

[17] Peterson BK, Weber JN, Kay EH, Fisher HS, Hoekstra HE (2021). Double digest RADseq: an inexpensive method for de novo SNP discovery and genotyping in model and non-model species. PloS one..

[18] Li H, Durbin R (2010). Fast and accurate long-read alignment with Burrows–Wheeler transform. Bioinformatics.

[19] Andrews, S. FastQC: A quality control tool for high throughput sequence data. http://www.bioinformatics.babraham.ac.uk/projects/fastqc (2010).

[20] Bushnell, Brian. BBMap: A Fast, Accurate, Splice-Aware Aligner. United States. https://www.osti.gov/servlets/purl/1241166 (2014).

[21] García-Alcalde F, Okonechnikov K, Carbonell J, Cruz LM, Götz S, Tarazona S, Dopazo J, Meyer TF, Conesa A (2012). Qualimap: Evaluating next-generation sequencing alignment data. Bioinformatics.

[22] Li H (2011). A statistical framework for SNP calling, mutation discovery, association mapping and population genetical parameter estimation from sequencing data. Bioinformatics.

[23] Vohra V, Chhotaray S, Gowane G, Alex R, Mukherjee A, Verma A, Deb SM (2021). Genome-wide association studies in Indian Buffalo revealed genomic regions for lactation and fertility. Front. Genet..

[24] Chang CC, Chow CC, Tellier LC, Vattikuti S, Purcell SM, Lee JJ (2015). Second-generation PLINK: Rising to the challenge of larger and richer datasets. Gigascience.

[25] Marees AT, de Kluiver H, Stringer S, Vorspan F, Curis E, Marie-Claire C, Derks EM (2018). A tutorial on conducting genome-wide association studies: Quality control and statistical analysis. Int. J. Methods Psychiatr. Res..

[26] Bush WS, Moore JH (2012). Chapter 11: Genome-wide association studies. PLoS Comput. Biol..

[27] Benjamini Y, Hochberg Y (1995). Controlling the false discovery rate: A practical and powerful approach to multiple testing. J. R. Stat. Soc. Ser. B Methodol..

[28] Barbosa AM (2015). fuzzySim: Applying fuzzy logic to binary similarity indices in ecology. Methods Ecol. Evol..

[29] Eldawy MH, Lashen MES, Badr HM, Farouk MH (2021). Milk production potential and reproductive performance of Egyptian buffalo cows. Trop. Anim. Health Prod..

[30] Choudhary S, Choudhary RK (2019). Rapid and efficient method of total RNA isolation from milk fat for transcriptome analysis of mammary gland. Proc. Natl. Acad. Sci. India Sect. B Biol. Sci..

[31] Batut, B. *et al.* Reference-based RNA-Seq data analysis (Galaxy Training Materials). https://training.galaxyproject.org/training-material/topics/transcriptomics/tutorials/ref-based/tutorial.html (Accessed 02 May 2022) (2022).

[32] Davey JW, Hohenlohe PA, Etter PD, Boone JQ, Catchen JM, Blaxter ML (2011). Genome-wide genetic marker discovery and genotyping using next-generation sequencing. Nat. Rev. Genet..

[33] Elshire RJ, Glaubitz JC, Sun Q, Poland JA, Kawamoto K, Buckler ES, Mitchell SE (2011). A robust, simple genotyping-by-sequencing (GBS) approach for high diversity species. PLoS ONE.

[34] Metzker ML (2010). Sequencing technologies—the next generation. Nat. Rev. Genet..

[35] Bhat SA, Ahmad SM, Ibeagha-Awemu EM, Bhat BA, Dar MA, Mumtaz PT, Shah RA, Ganai NA (2019). Comparative transcriptome analysis of mammary epithelial cells at different stages of lactation reveals wide differences in gene expression and pathways regulating milk synthesis between Jersey and Kashmiri cattle. PLoS ONE.

[36] Bai WL, Yin RH, Jiang WQ, Ajayi OO, Zhao SJ, Luo GB, Zhao ZH, Imumorin IG (2013). Molecular analysis of αs1-, β-, αs2-and κ-casein transcripts reveals differential translational efficiency in yak lactating mammary gland. Livest. Sci..

[37] Boutinaud M, Herve L, Lollivier V (2015). Mammary epithelial cells isolated from milk are a valuable, non-invasive source of mammary transcripts. Front. Genet..

[38] Parrish RL, Gibson GC, Epstein MP, Yang J (2022). TIGAR-V2: Efficient TWAS tool with nonparametric Bayesian eQTL weights of 49 tissue types from GTEx V8. Hum. Genet. Genomics Adv..

[39] Zeng P, Zhou X (2017). Non-parametric genetic prediction of complex traits with latent Dirichlet process regression models. Nat. Commun..

[40] Ye M, Xu M, Lu M, Zhou B, El-Kader HA, Alam SS, Mahrous KF (2020). Identification of candidate genes associated with milk yield trait in buffaloes (*Bubalus bubalis*) by restriction-site-associated DNA sequencing. Revista Brasileira de Zootecnia.

[41] Rezaei R, Wu Z, Hou Y, Bazer FW, Wu G (2016). Amino acids and mammary gland development: Nutritional implications for milk production and neonatal growth. J. Anim. Sci. Biotechnol..

[42] Wathes DC, Cheng Z, Salavati M, Buggiotti L, Takeda H, Tang L, Becker F, Ingvartsen KI, Ferris C, Hostens M, Crowe MA, GplusE Consortium (2021). Relationships between metabolic profiles and gene expression in liver and leukocytes of dairy cows in early lactation. J. Dairy Sci..

[43] Ahlawat S, Arora R, Sharma U, Sharma A, Girdhar Y, Sharma R, Kumar A, Vijh RK (2021). Comparative gene expression profiling of milk somatic cells of Sahiwal cattle and Murrah buffaloes. Gene.

[44] Liu S, Ye T, Li Z, Li J, Jamil AM, Zhou Y, Hua G, Liang A, Deng T, Yang L (2019). Identifying hub genes for heat tolerance in water buffalo (*Bubalus bubalis*) using transcriptome data. Front. Genet..

[45] Nadeem A, Maryam J (2016). Genetic and genomic dissection of Prolactin revealed potential association with milk production traits in riverine buffalo. Trop. Anim. Health Prod..

[46] Parmentier I, Portetelle D, Gengler N, Prandi A, Bertozzi C, Vleurick L, Gilson R, Renaville R (1999). Candidate gene markers associated with somatotropic axis and milk selection. Domest. Anim. Endocrinol..

[47] El-Komy SM, Saleh AA, Abdel-Hamid TM, El-Magd MA (2020). Association of ghr polymorphisms with milk production in buffaloes. Animals.

[48] Wu Z, Tian M, Heng J, Chen J, Chen F, Guan W, Zhang S (2020). Current evidences and future perspectives for AMPK in the regulation of milk production and mammary gland biology. Front. Cell Dev. Biol..

[49] Wu Z, Li Q, Yang S, Zheng T, Shao J, Guan W, Chen F, Zhang S (2022). Energy deprivation-induced AMPK activation inhibits milk synthesis by targeting PrlR and PGC-1α. Cell Commun. Signal..

[50] Huang J, Guesthier MA, Burgos SA (2020). AMP-activated protein kinase controls lipid and lactose synthesis in bovine mammary epithelial cells. J. Dairy Sci..

[51] Du C, Deng TX, Zhou Y, Ghanem N, Hua GH (2020). Bioinformatics analysis of candidate genes for milk production traits in water buffalo (*Bubalus bubali*s). Trop. Anim. Health Prod..

[52] Yang SQ, Chen YD, Li H, Hui X, Gao WY (2018). Geniposide and gentiopicroside suppress hepatic gluconeogenesis via regulation of AKT-FOXO1 pathway. Arch. Med. Res..

[53] Jacometo CB, Zhou Z, Luchini D, Trevisi E, Corrêa MN, Loor JJ (2016). Maternal rumen-protected methionine supplementation and its effect on blood and liver biomarkers of energy metabolism, inflammation, and oxidative stress in neonatal Holstein calves. J. Dairy Sci..

[54] Zhou C, Shen D, Li C, Cai W, Liu S, Yin H, Shi S, Cao M, Zhang S (2019). Comparative transcriptomic and proteomic analyses identify key genes associated with milk fat traits in Chinese Holstein cows. Front. Genet..

[55] Li N, Zhao F, Wei C, Liang M, Zhang N, Wang C, Li QZ, Gao XJ (2014). Function of SREBP1 in the milk fat synthesis of dairy cow mammary epithelial cells. Int. J. Mol. Sci..

[56] Khan MZ, Khan A, Xiao J, Ma Y, Ma J, Gao J, Cao Z (2020). Role of the JAK-STAT pathway in bovine mastitis and milk production. Animals.

[57] Sigl T, Meyer HHD, Wiedemann S (2014). Gene expression analysis of protein synthesis pathways in bovine mammary epithelial cells purified from milk during lactation and short-term restricted feeding. J. Anim. Physiol. Anim. Nutr..

[58] Ji MR, Lee SI, Jang YJ, Jeon MH, Kim JS, Kim KW, Park JK, Yoo JG, Jeon IS, Kwon DJ, Park CK (2015). STAT5 plays a critical role in regulating the 5′-flanking region of the porcine whey acidic protein gene in transgenic mice. Mol. Reprod. Dev..

[59] Zhou M, Xu L, Zhao F, Liu H (2021). Regulation of milk protein synthesis by free and peptide-bound amino acids in dairy cows. Biology.

