# Supplementary material for: TWAS revealed significant causal loci for milk production and its composition in Murrah buffaloes
Source: Sci Rep. 2023 Dec 16;13:22401. doi: 10.1038/s41598-023-49767-x (PMC10725422; doi:10.1038/s41598-023-49767-x)
Supplement: Supplementary file 1 — Supplementary Information 1. [file 41598_2023_49767_MOESM1_ESM.docx]

**Supplementary file for methods**

***Generation of genotype information***

Blood sample from 144 randomly selected animals were collected aseptically and DNA was isolated via Phenol-Chloroform method following protocol of Sambrook and Russell (2006). DNA of the samples were outsourced for genotyping by ddRAD sequencing technology using Illumina HiSeq 2000 platform. Quality of DNA was checked using agarose gel electrophoresis and quantity was assessed using Qubit 4.0 fluorometer. ddRAD-Seq was performed using Sphl and Mluc1 Restriction enzymes to cleave genomic DNA into fragments. It uses precise size selection to choose the best fragments for sequencing. Only fragments cut with both enzymes and of the appropriate size were sequenced. Cleanup of the digested product was done using Ampure beads. Adaptors were ligated using T4 DNA ligase and ligated products were cleaned and pooled up. Size selection of the product was done after 2% agarose gel electrophoresis. PCR amplification was performed to enrich and add the Illumina specific adapters and flow cell annealing sequences. Bio analyzer was used to check the quality of library. Final pooling and sequencing was done to obtain raw fastq reads.

***Variant calling from ddRAD sequencing data***

Index and sequence dictionary files for reference genome retrieved from NCBI website were created using the Burrows–Wheeler algorithm (BWA) (Li and Durbin, 2010) and PicardTools, respectively. The quality of paired-end raw FASTQ files generated after sequencing was checked using FastQC (Andrews, 2010), and each report was combined through MultiQC (Ewels et al., 2016). Adapters were marked and trimmed using bbmap (Brian, 2014). The BWA-MEM algorithm was used to align the trimmed FASTQ sequences with the reference genome. Aligned files were coordinate-sorted, and duplicate reads were removed. Read group identifiers were updated using PicardTools. The quality of aligned BAM files was checked using qualimap (García-Alcalde et al., 2012). Variants were called using bcftools-mpileup (Li, 2011). We standardized this variant calling from pipeline (Vohra et al., 2021) (Fig. 3.1) and two sets of variant calling were performed. Set-I variants were called based on the latest Murrah buffalo reference genome GCF_019923935.1_NDDB_SH_1_genomic.fna and variants were retained for further training of the dataset to predict eQTL weights and individual level transcriptome wide association study. A second set (Set-II) of variants were called based on the Mediterranean buffalo reference genome GCF_003121395.1_ASM312139v1_genomic.fna.

***Genome-wide association study (GWAS) using the Set-II variants***

Genotype data were further processed in PLINK v1.9 for quality control (Table 1) before performing GWAS. QC passed data were further used for GWAS on listed lactation traits in buffaloes. GWAS was conducted for each trait separately regressing genotypes upon traits.

**Table 1: Quality control parameters to be checked**

| Parameters | Values |
| --- | --- |
| Genotype call rate | > 95% |
| LD pruning | r2 < 0.8 |
| Hardy-Weinberg Equilibrium | p<0.0001 |
| MAF | > 0.05 |
| Autosomes + X | To be retained |

Genome-wide identity-by-state (IBS) for all pairs of individuals was checked. Multidimensional scaling (MDS) based on SNP information was done to check for the presence of any population stratification and was corrected by incorporating the first two MDS components as covariates in the model for GWAS. Birth weight (bwt) and age at first calving in months (AFC) were also included as covariates in the model. A genome-wide scan for significant SNPs considering only additive effects was accomplished through a simple regression model using PLINK v1.9 as described by Marees et al. (2018), where residuals were assumed to be normally and independently distributed. A linear regression model was fitted for determining the association between SNPs and continuous traits (Bush and Moore, 2012). The threshold for genome-wide significance was determined by correcting the *P*-values of the SNP association test with Bonferroni’s correction and was 1.28$\times$10^-6^. *P*-values of the top ten SNPs of each GWAS traits was corrected for Benjamini–Hochberg’s false discovery rate (FDR) at 5% levels (Benjamini and Hochberg, 1995) using the “R” package fuzzySim v3.0 (Barbosa, 2020) and is given in the supplementary document. The results were plotted as Manhattan plots and Q-Q plots using the “qqman” package of R.

*Linear regression model used for GWAS:*

***y* = β****_0_ +** ***x******β****_1_ +** **C****_1_*****β****_2_ +** **C****_2_*****β****3 +** **AFC*β_4_ + bwt*β_5_ + e**

where, *y* = trait, *x* = additive effect of SNPs, C1 = first component of MDS, C2 = second component of MDS, AFC = Age at first calving in months, bwt = birth weight of the animal (in Kgs), β0 = intercept term, β1 = regression coefficient representing the strength of association between SNP *x* and trait *y*, β2 = regression coefficient of C1, β3 = regression coefficient of C2, β4 = regression coefficient of AFC, β5 = regression coefficient of bwt, and *e* = residuals or noise not explained by SNPs.

***Generation of Gene expression information***

For integrating transcriptomic information to find the underlying gene significantly contributing to the expression of complex lactational traits, 8 animals in milk (mid lactation stage) from those having both genotype and phenotype data were selected. Animals were divided into two groups namely high yielder (>2400 kg/lactation) and low yielder (<1800 kg/lactation). Approximately, 150-200 ml of milk was collected aseptically in DEPC treated tubes. RNA isolation was performed under sterile conditions in lab. Presence of RNase in working area and equipment was checked using RNAseAWAY. RNA was isolated following the protocol of Choudhary and Choudhary (2018).

***Isolation of epithelial cell crescents from milk***

1. Milk samples were centrifuged at 1000 ×g for 20 mins at 4^0^C to pellet the somatic cells and separate milk fat layer.
2. The upper layer of fat was removed by a sterile spatula and transferred into a new 15 ml tube.
3. The fat layer was resuspended in 1.5 ml of trizol/500 ng milk fat in a 50 ml centrifuge tube.
4. The mixture was vortexed and then put in ice for 5 minutes.
5. Tubes containing the mixture were centrifuged at 12000 rpm for 10 min at 4 ^0^C to remove lipids and the liquid phase obtained were transferred into new 2 ml tubes.

***RNA isolation from mammary epithelial cell crescents***

1. 1ml aliquot of cells + trizol mixture was put in a 2 ml tube.
2. Chloroform (200 µl) was added to the same.
3. The mixture was centrifuged at 10,000 rpm for 10 min at 4 ^0^C.
4. After centrifugation, supernatant was decanted to another 1.5 ml tube.
5. Equal volume of chilled Isopropanol was added.
6. It was mixed and kept on ice for 15 minutes.
7. RNA was isolated using RNAeasy mini kit (only RPE and RW1 buffer were used).
8. The mixture was transferred into 2 ml RNAeasy mini spin column already provide in kit.
9. Spin columns were centrifuged at 8000 rpm for 2 minutes
10. The flow-through liquid was discarded as RNA is on the column now.
11. 700 µl of Buffer RW1 was added to RNeasy spin column.
12. The spin column was centrifuged at 2 minutes at 8000 rpm.
13. The flow-through liquid was discarded.
14. 700 µl of Buffer RPE was added to RNeasy spin column.
15. The spin columns were centrifuged at 2 minutes at 8000 rpm.
16. The flow-through liquid was discarded.
17. The steps 14-16 were repeated again and once 15-16 were repeated without any wash buffer.
18. RNA was eluted in 30 µl of RNAse free water after transferring the column to a 1.5 ml nuclease free centrifuge tube.
19. The tube containing spin columns were centrifuged again to collect RNA.

***RNA quality check***

Extracted RNA quantity was checked on Qubit 4.0 fluorometer (Thermofisher #Q33238) using RNA HS assay kit (Thermofisher #Q32851) following manufacturer’s protocol. To measure the purity of the extraction we also measure the concentration on Nanodrop 1000. Finally, to obtain RIN values RNA was checked on the Tapestation using HS RNA screentape (Agilent).

***mRNA Enrichment and Library Preparation***

250ng of Total RNA was used to enrich the mRNA using NEBNext Poly (A) mRNA magnetic isolation module (Catalog: E7490, New England Biolabs) by following the manufacturers’ protocol. The enriched mRNAs were further taken for the library preparation using the NEBNext® Ultra™ II RNA Library Prep Kit for Illumina (Catalog: E7775S, New England Biolabs). In brief, the enriched mRNAs were primed with NEBNext Random Primers and chemically fragmented in a magnesium-based buffer at 94°C for 10 minutes in order to get an inserts of ~200 nucleotides. The fragmented mRNAs were reverse transcribed to form cDNA and the first strand cDNA reactions were converted to dS DNA. The double stranded cDNA fragments obtained were cleaned up by using 1.8X of AMPure XP beads (Catalog: A63881, Beckman Coulter). The cDNA undergo end repair where in the mix converts the overhangs resulting from fragmentation into blunt ends. The 3’ to 5’ exonuclease activity of end repair mix removes the 3’ overhangs and polymerase activity fills in the 5’ overhangs. To the blunt ended fragments, adenylation was performed by adding single ‘A’ nucleotide to the 3’ ends. To the adenylated fragments, loop adapters were ligated and cleaved with uracil-specific excision reagent (USER) enzyme Size Selection was performed using AMPure beads (Catalog: A63881, Beckman Coulter) with manufacturer’s protocol aiming the library size of 400-600bp. Furthermore, the cDNA was amplified by 12 cycles of PCR with the addition of NEBNext Ultra II Q5 master mix, and “NEBNext® Multiplex Oligos for Illumina” to facilitate multiplexing while sequencing. The amplified products were then purified using 0.9X AMPure XP beads (Catalog: A63881, Beckman Coulter) and the final library was eluted in 15µl of 0.1X TE buffer. Final libraries were quantified using Qubit 4.0 fluorometer (Thermofisher #Q33238) using DNA HS assay kit (Thermofisher #Q32851) following manufacturer’s protocol. To identify the insert size of the library, Tapestation 4150 (Agilent) utilizing high sensitive D1000 screentapes (Agilent # 5067- 5582) was used following manufacturers’ protocol.

***RNAseq data analysis***

RNAseq data analysis was performed following the workflow of (Batut et al., 2022, Reference-based RNA-Seq data analysis (Galaxy Training Materials). Online; accessed Mon May 02 2022).


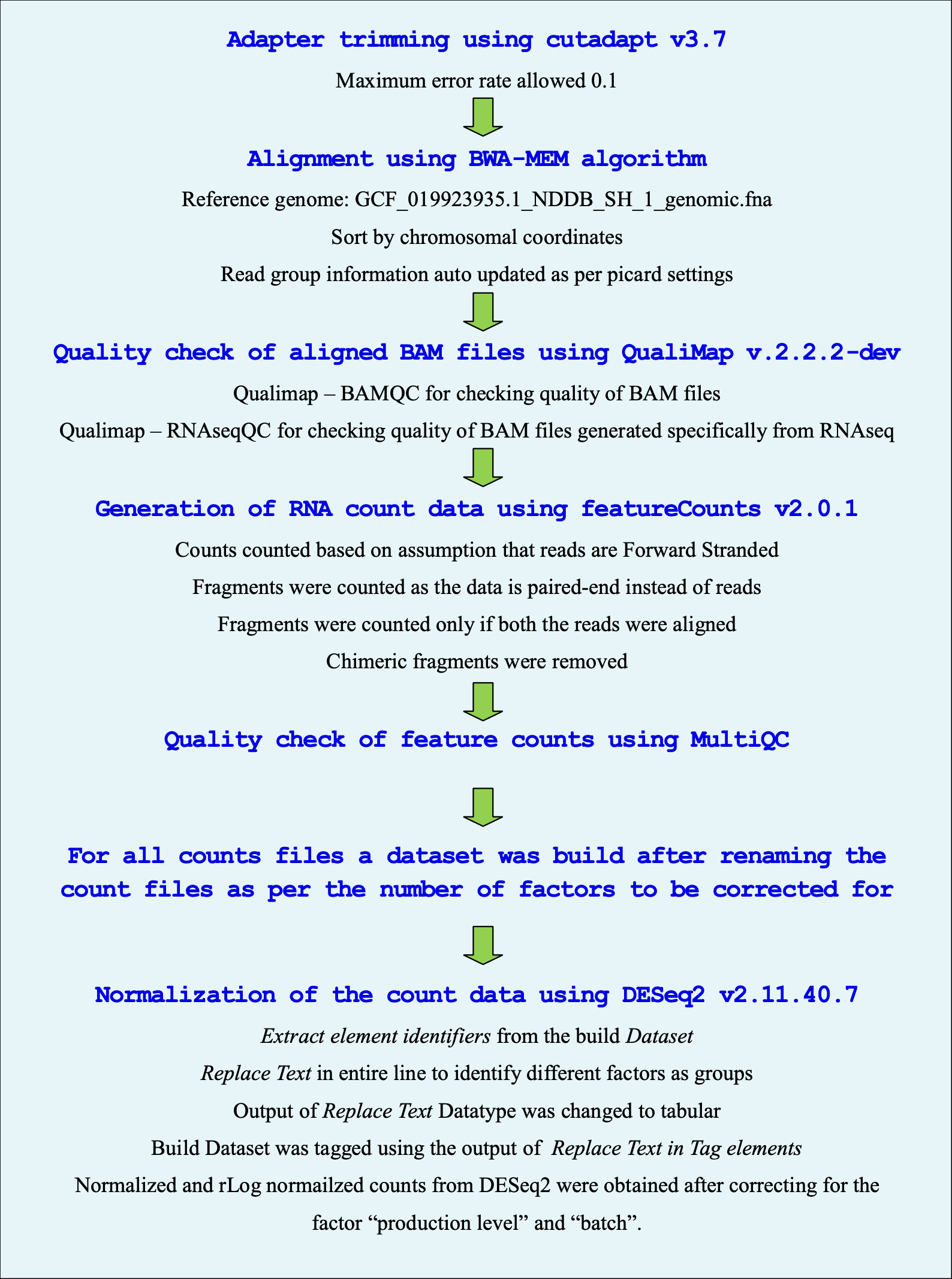


***Transcriptome-wide association study***

To perform a two stage TWAS, first gene expression imputation model was designed for estimating the cis-eQTL effect sizes from a training sample (n=8) for which both genotype and transcriptome data are available. The following model suggested by Nagpal et al. (2019) was employed:

**E_g_ = *X*_train_w +** **ε, ε ~ *N*(0, σ_ε_^2^*I*) --------------**eq.1

Where,

**E_g_**: denotes the log normalized gene expression levels (after corrections for confounding factors such as production levels and sampling batch) for gene g

***X*_train_**: denotes the genotype matrix for all cis-genotypes (encoded as the number of minor alleles present 1MB of the gene; [-1 MB—Gene_start—Gene_end—+1 MB])

**w**: denotes the corresponding cis-eQTL effect-size vector, and

**ε**: denotes the error term

The gene expression levels GReX (genetically regulated gene expression) of the test samples (n=136) were imputed with the assumption of following model:

$\overset{^}{\boldsymbol{GReX}}$ **= *X*_test_**$\overset{^}{\boldsymbol{w}}$ **------------------------------**eq.2

Given the predicted eQTL effect size estimates $\overset{^}{w}$from the training data in eq.1, *GReX* was imputed by the eq.2 where *X***_test_** is the genotype matrix containing cis-SNP data for the test dataset.

***Nonparametric Bayesian method***

The nonparametric Bayesian Dirichlet Process Regression (DPR) model with assumption of a normal prior ***N*(0, σ_w_^2^)** for the cis-eQTL effect sizes (w_i_, i = 1,....,p) and a Dirichlet Process prior for the effect size variances **σ_w_^2^** was used for the model training and estimation of cis-eQTL effect sizes i.e., **w_i_** ~ ***N*(0, σ_w_^2^), σ_w_^2^ ~ D, D ~ DP(IG(a, b), ξ)**. Where, **ξ** is the concentration parameter, and a non-informative prior is set for the hyper-parameters **a_k_**, **b_k_**, **a_ξ_**, and **b_ξ_** as 0.1 in the inverse gamma distribution and (**a_ξ_**, **b_ξ_**) was set as (1, 0.1) in the gamma distribution. The posterior estimates for **w** were obtained by the MCMC algorithm.

***Parametric Elastic-Net method***

The Elastic-Net model (Zou and Hastie, 2005) is a sparse form of penalised regression that estimates the cis-eQTL effect sizes ($\overset{^}{\boldsymbol{w}}$) with a combination of Lasso (L_1_) and Ridge (L_2_) penalties. Elastic-Net uses a penalty parameter **λ** and tunes it by assuming **α** = 0.5, where **α** denotes proportion of L_1_ penalty.

***Association study with TIGAR***

TIGAR: An Improved Bayesian Tool for Transcriptomic Data Imputation Enhances Gene Mapping of Complex Traits was used for association analysis. Several simulations were run to test the appropriate parameters for the training model. Training was done with 5-fold cross validation and without cross validation (leaving two out of 8 samples rotationally per iteration and training with all 8 samples) for both DPR and Elastic-Net model. Only fixed effects of the cis-SNPs on genes were estimated as random effects tend to be overestimated in the small training samples, and estimation of only fixed effects provides better prediction accuracy. SNPs were excluded if missing rate exceeded 0.2. Those SNPs having a MAF<0.01 and deviating from the Hardy-Weinberg Equilibrium at p<0.0001 were also excluded from the training. For training and individual level associations Set-I variants were used.

***TWAS with univariate phenotype***

**f(E[Y|X,C]) = ηC + β**$\overset{^}{\boldsymbol{GReX}}$ ------------------------------eq.3

Where,

f(.) is a pre-specified link function, which is set as identity function for the quantitative phenotype

[Y|X,C]: Phenotype given genotype matrix *X*_test_ and covariate matrix C

H_0_ : β = 0 in eq.3

Same covariates as that of GWAS i.e., AFC, Birth weight, and 1^st^ two components of MDS of set-II variants were taken to maintain homogeneity. TWAS was performed for 305 DMY, PY, WA, FP and SNFP in the test individuals (*N*=136).

***Comparison of GWAS and TWAS results***

Chromosome wise TWAS results for each trait and each model were combined to generate TWAS Manhattan plots. Manhattan plots generated from GWAS for each trait were compared with the TWAS Manhattan plots. The significant genes and peak signals from the TWAS results were noted for further analysis.

***Gene ontology and enrichment analysis***

The peak signals from the TWAS results obtained via the DPR method were considered to have important role in regulating the studied lactational traits. For underlining biological role of genes present in such regions, all the genes ±3 Mb of the above mentioned regions were extracted for all the studies traits and a combined gene ontology and network enrichment analysis was conducted to identify trait specific novel genes through the online gene ontology analysis platform gProfiler.
