# Supplementary material for: TWAS revealed significant causal loci for milk production and its composition in Murrah buffaloes
Source: Sci Rep. 2023 Dec 16;13:22401. doi: 10.1038/s41598-023-49767-x (PMC10725422; doi:10.1038/s41598-023-49767-x)
Supplement: Supplementary file 2 — Supplementary Information 2. [file 41598_2023_49767_MOESM2_ESM.docx]

**Supplementary file:**

**Table S1: Quality control thresholds and variants filtered in the subsequent steps**

| **Parameters** | **Threshold Values** | **Variants removed (Set-I)** | **Variants removed (Set-II)** |
| --- | --- | --- | --- |
| Multiallelic and duplicate sites | To be removed completely | 1,22,659 | 1,21,804 |
| Genotype call rate | > 95% | 52,38,807 | 52,53,204 |
| MAF | > 0.01 (Set-I)  > 0.05 (Set-II) | 1,44,554 | 2,63,557 |
| Hardy-Weinberg Equilibrium | p<0.0001 | 1,08,845 | 1,12,205 |
| SNPs Only | All indels to be removed | 12,487 | 6,146 |
| LD pruning | r2 < 0.8 | - | 8,601 |
| Non-autosomal and non-X chromosomal | To be removed completely | - | 157 |
| Total Variants removed in QC |  | 56,27,352 | 5,765,674 |

**Table S2: Top 10 genes and SNPs having lowest *P*-value in TWAS and GWAS with the 305 days milk yield, respectively**

| **Chr** | **SNP/Gene Name** | ***P*-value** | **FDR** | **Method** |
| --- | --- | --- | --- | --- |
| 11 | NC_037555.1:30281597[MU] | 8.46×10^-06^ | 0.33 | GWAS |
| 16 | NC_037560.1:47885286[MU] | 4.00×10^-05^ | 0.422 |  |
| 10 | NC_037554.1:19195382[MU] | 5.04×10^-05^ | 0.422 |  |
| 3 | NC_037547.1:88029567[MU] | 5.41×10^-05^ | 0.422 |  |
| 3 | NC_037547.1:152863609[MU] | 5.41×10^-05^ | 0.422 |  |
| 17 | NC_037561.1:29702216[MU] | 6.77×10^-05^ | 0.4401 |  |
| 20 | NC_037564.1:13906580[MU] | 9.61×10^-05^ | 0.4853 |  |
| 16 | NC_037560.1:74362826[MU] | 9.95×10^-05^ | 0.4853 |  |
| 24 | NC_037568.1:36865371[MU] | 0.000122 | 0.5189 |  |
| 2 | NC_037546.1:18564231[MU] | 0.000135 | 0.5189 |  |
| 4 | *RYR2* | 1.57×10^-05^ | 0.358686 | EN_NOCV |
| 8 | *LOC123334652* | 4.64×10^-05^ | 0.45429 |  |
| 12 | *LOC102412448* | 9.40×10^-05^ | 0.45429 |  |
| 6 | *LOC123334086* | 0.000127 | 0.45429 |  |
| 11 | *LOC102405757* | 0.000176 | 0.45429 |  |
| 2 | *METTL21A* | 0.000194 | 0.45429 |  |
| 23 | *LGI1* | 0.000201 | 0.45429 |  |
| 4 | *LMO3* | 0.000205 | 0.45429 |  |
| 13 | *TRNAC-ACA_139* | 0.000221 | 0.45429 |  |
| 15 | *MAF1* | 0.000248 | 0.45429 |  |
| 9 | *NDUFA11* | 6.02×10^-06^ | 0.132973 | EN_CV |
| 21 | *ARPC4* | 3.29×10^-05^ | 0.256558 |  |
| 6 | *LOC102409536* | 3.48×10^-05^ | 0.256558 |  |
| 24 | *LOC102403559* | 9.59×10^-05^ | 0.324817 |  |
| 12 | *TTC27* | 9.81×10^-05^ | 0.324817 |  |
| 5 | *LOC123465808* | 0.000101 | 0.324817 |  |
| 17 | *IL15* | 0.000103 | 0.324817 |  |
| 23 | *LOC102399316* | 0.000158 | 0.343608 |  |
| 12 | *MOGS* | 0.000168 | 0.343608 |  |
| 3 | *CBX1* | 0.000173 | 0.343608 |  |
| 10 | *LOC112587322* | 1.31×10^-05^ | 0.354222 | DPR_NOCV |
| 10 | *LOC112587321* | 2.73×10^-05^ | 0.367728 |  |
| 16 | *LOC112579753* | 9.80×10^-05^ | 0.449224 |  |
| 12 | *TMEM178A* | 0.000125 | 0.449224 |  |
| 14 | *STAM* | 0.000135 | 0.449224 |  |
| 7 | *LOC112582652* | 0.000147 | 0.449224 |  |
| 3 | *LOC102396194* | 0.000154 | 0.449224 |  |
| 20 | *LOC123330713* | 0.000177 | 0.449224 |  |
| 7 | *TRNA×10-UUC_83* | 0.00023 | 0.449224 |  |
| 8 | *THAP5* | 0.000252 | 0.449224 |  |

*Note: Chr – Chromosome no., FDR – False discovery rate corrected P-value, EN_NOCV – Elastic Net model without cross-validation, EN_CV – Elastic Net model with cross-validation, and DPR_NOCV – DPR model without cross-validation*

**Table S3: Top 10 genes and SNPs having lowest *P*-value in TWAS and GWAS with the peak yield, respectively**

| **Chr** | **SNP/Gene Name** | ***P*-value** | **FDR** | **Method** |
| --- | --- | --- | --- | --- |
| 24 | NC_037568.1:28499463[MU] | 7.92×10^-06^ | 0.1576 | GWAS |
| 20 | NC_037564.1:13906580[MU] | 8.08×10^-06^ | 0.1576 |  |
| 4 | NC_037548.1:44518782[MU] | 1.89×10^-05^ | 0.2066 |  |
| 3 | NC_037547.1:152863609[MU] | 2.12×10^-05^ | 0.2066 |  |
| 11 | NC_037555.1:30281597[MU] | 3.94×10^-05^ | 0.287 |  |
| 11 | NC_037555.1:24583445[MU] | 4.87×10^-05^ | 0.287 |  |
| 2 | NC_037546.1:18564231[MU] | 5.85×10^-05^ | 0.287 |  |
| 10 | NC_037554.1:16657423[MU] | 5.89×10^-05^ | 0.287 |  |
| 10 | NC_037554.1:17324461[MU] | 7.10×10^-05^ | 0.3037 |  |
| 23 | NC_037567.1:32648766[MU] | 8.55×10^-05^ | 0.3037 |  |
| 2 | *SSR1* | 3.98×10^-06^ | 0.091269 | EN_NOCV |
| 6 | *LOC123334086* | 2.03×10^-05^ | 0.20285 |  |
| 24 | *LOC123331613* | 3.21×10^-05^ | 0.20285 |  |
| 9 | *LONP1* | 3.54×10^-05^ | 0.20285 |  |
| 9 | *RLN3* | 4.65×10^-05^ | 0.212824 |  |
| 19 | *TRNAR-UCG_5* | 5.95×10^-05^ | 0.227107 |  |
| 10 | *LATS1* | 7.20×10^-05^ | 0.235562 |  |
| 11 | *PALS1* | 8.34×10^-05^ | 0.238742 |  |
| 21 | *TKT* | 0.000113 | 0.287804 |  |
| 24 | *LOC102394449* | 0.000127 | 0.290193 |  |
| 9 | *NDUFA11* | 4.64×10^-06^ | 0.102442 | EN_CV |
| 4 | *LOC112584712* | 2.48×10^-05^ | 0.146642 |  |
| 24 | *LOC123331613* | 3.21×10^-05^ | 0.146642 |  |
| 11 | *VCPKMT* | 3.75×10^-05^ | 0.146642 |  |
| 6 | *FUBP1* | 4.58×10^-05^ | 0.146642 |  |
| 12 | *CTNNA2* | 4.61×10^-05^ | 0.146642 |  |
| 9 | *RLN3* | 4.65×10^-05^ | 0.146642 |  |
| 19 | *TRNAR-UCG_5* | 5.73×10^-05^ | 0.158112 |  |
| 11 | *PALS1* | 8.31×10^-05^ | 0.201978 |  |
| 5 | *LOC102406725* | 9.14×10^-05^ | 0.201978 |  |
| 3 | *TRNAW-CCA_36* | 4.33×10^-06^ | 0.040194 | DPR_NOCV |
| 9 | *LOC102411990* | 7.46×10^-06^ | 0.040194 |  |
| 9 | *LOC102415512* | 7.46×10^-06^ | 0.040194 |  |
| 9 | *LOC102416173* | 7.46×10^-06^ | 0.040194 |  |
| 9 | *LOC123335276* | 7.46×10^-06^ | 0.040194 |  |
| 20 | *PPP4R4* | 5.01×10^-05^ | 0.22527 |  |
| 15 | *EIF3E* | 8.61×10^-05^ | 0.325597 |  |
| 20 | *LOC123330700* | 9.66×10^-05^ | 0.325597 |  |
| 20 | *SERPINA11* | 0.000189 | 0.456904 |  |
| 24 | *LOC112581867* | 0.000192 | 0.456904 |  |

*Note: Chr – Chromosome no., FDR – False discovery rate corrected P-value, EN_NOCV – Elastic Net model without cross-validation, EN_CV – Elastic Net model with cross-validation, and DPR_NOCV – DPR model without cross-validation*

**Table S4: Top 10 genes and SNPs having lowest *P*-value in TWAS and GWAS with the wet average, respectively**

| **Chr** | **SNP/Gene Name** | ***P*-value** | **FDR** | **Method** |
| --- | --- | --- | --- | --- |
| 4 | NC_037548.1:44518782[MU] | 5.97×10^-06^ | 0.1092 | GWAS |
| 18 | NC_037562.1:37277083[MU] | 6.12×10^-06^ | 0.1092 |  |
| 3 | NC_037547.1:88029567[MU] | 8.40×10^-06^ | 0.1092 |  |
| 10 | NC_037554.1:48419016[MU] | 1.36×10^-05^ | 0.1284 |  |
| 6 | NC_037550.1:112228435[MU] | 1.97×10^-05^ | 0.1284 |  |
| 24 | NC_037568.1:28499463[MU] | 1.97×10^-05^ | 0.1284 |  |
| 10 | NC_037554.1:16657423[MU] | 2.71×10^-05^ | 0.151 |  |
| 6 | NC_037550.1:112228371[MU] | 3.85×10^-05^ | 0.1684 |  |
| 11 | NC_037555.1:30281597[MU] | 4.30×10^-05^ | 0.1684 |  |
| 21 | NC_037565.1:39649505[MU] | 4.56×10^-05^ | 0.1684 |  |
| 2 | *METTL21A* | 1.83×10^-05^ | 0.196299 | EN_NOCV |
| 4 | *RYR2* | 1.91×10^-05^ | 0.196299 |  |
| 8 | *LOC123334652* | 2.57×10^-05^ | 0.196299 |  |
| 10 | *LATS1* | 3.90×10^-05^ | 0.223071 |  |
| 9 | *NLRP3* | 8.63×10^-05^ | 0.395113 |  |
| 9 | *LONP1* | 0.000113 | 0.431046 |  |
| 12 | *MRPL30* | 0.000195 | 0.464538 |  |
| 15 | *MFSD3* | 0.0002 | 0.464538 |  |
| 23 | *LOC123331343* | 0.00029 | 0.464538 |  |
| 8 | *TSGA13* | 0.000318 | 0.464538 |  |
| 2 | *METTL21A* | 1.82×10^-05^ | 0.223712 | EN_CV |
| 9 | *NDUFA11* | 2.24×10^-05^ | 0.223712 |  |
| 14 | *LOC123329228* | 3.04×10^-05^ | 0.223712 |  |
| 7 | *LOC112586189* | 6.37×10^-05^ | 0.324998 |  |
| 21 | *ARPC4* | 9.41×10^-05^ | 0.324998 |  |
| 4 | *LOC112584712* | 0.000101 | 0.324998 |  |
| 2 | *LOC123332232* | 0.000111 | 0.324998 |  |
| 15 | *SHARPIN* | 0.000125 | 0.324998 |  |
| 4 | *RET* | 0.000132 | 0.324998 |  |
| 3 | *SLC24A2* | 0.000151 | 0.334605 |  |
| 17 | *LOC112579963* | 5.71×10^-05^ | 0.450883 | DPR_NOCV |
| 11 | *CTXN2* | 0.000119 | 0.450883 |  |
| 18 | *PSME3IP1* | 0.000121 | 0.450883 |  |
| 20 | *LOC123330713* | 0.000169 | 0.450883 |  |
| 2 | *CNR2* | 0.000197 | 0.450883 |  |
| 5 | *CCNL2* | 0.00021 | 0.450883 |  |
| 15 | *LOC112579064* | 0.000246 | 0.450883 |  |
| 21 | *LOC123331013* | 0.000259 | 0.450883 |  |
| 8 | *LOC102399397* | 0.000265 | 0.450883 |  |
| 15 | *LOC112577757* | 0.000269 | 0.450883 |  |

*Note: Chr – Chromosome no., FDR – False discovery rate corrected P-value, EN_NOCV – Elastic Net model without cross-validation, EN_CV – Elastic Net model with cross-validation, and DPR_NOCV – DPR model without cross-validation*

**Table S5: Top 10 genes and SNPs having lowest *P*-value in TWAS and GWAS with the Fat%, respectively**

| **Chr** | **SNP/Gene Name** | ***P*-value** | **FDR** | **Method** |
| --- | --- | --- | --- | --- |
| 4 | NC_037548.1:48399143[MU] | 3.34×10^-06^ | 0.0691 | GWAS |
| 3 | NC_037547.1:162275901[MU] | 4.49×10^-06^ | 0.0691 |  |
| 5 | NC_037549.1:84739212[MU] | 8.15×10^-06^ | 0.0691 |  |
| 17 | NC_037561.1:15895247[MU] | 8.49×10^-06^ | 0.0691 |  |
| 22 | NC_037566.1:15166346[MU] | 8.86×10^-06^ | 0.0691 |  |
| 8 | NC_037552.1:58357861[MU] | 1.58×10^-05^ | 0.09611 |  |
| 3 | NC_037547.1:91898446[MU] | 1.72×10^-05^ | 0.09611 |  |
| 4 | NC_037548.1:40204848[MU] | 2.08×10^-05^ | 0.09761 |  |
| 20 | NC_037564.1:34215807[MU] | 2.25×10^-05^ | 0.09761 |  |
| 16 | NC_037560.1:66495537[MU] | 2.70×10^-05^ | 0.1055 |  |
| 4 | *SCUBE1* | 1.37×10^-05^ | 0.177459 | EN_NOCV |
| 13 | *SLC25A15* | 1.55×10^-05^ | 0.177459 |  |
| 8 | *RBM33* | 3.30×10^-05^ | 0.221156 |  |
| 1 | *LOC112587424* | 3.86×10^-05^ | 0.221156 |  |
| 2 | *LOC112579546* | 7.35×10^-05^ | 0.258089 |  |
| 6 | *BMP8B* | 8.19×10^-05^ | 0.258089 |  |
| 3 | *TTLL6* | 9.68×10^-05^ | 0.258089 |  |
| 8 | *IMMP2L* | 0.000103 | 0.258089 |  |
| 4 | *LOC123465659* | 0.000118 | 0.258089 |  |
| 3 | *LOC123465614* | 0.000128 | 0.258089 |  |
| 22 | *ARHGAP28* | 1.75×10^-05^ | 0.271569 | EN_CV |
| 13 | *LOC123328889* | 2.46×10^-05^ | 0.271569 |  |
| 1 | *LOC112587424* | 3.86×10^-05^ | 0.28424 |  |
| 6 | *LOC123334275* | 6.64×10^-05^ | 0.299438 |  |
| 10 | *IYD* | 6.78×10^-05^ | 0.299438 |  |
| 1 | *SMCO1* | 0.000178 | 0.333142 |  |
| 16 | *LOC123329732* | 0.000191 | 0.333142 |  |
| 3 | *EVI2A* | 0.000192 | 0.333142 |  |
| 7 | *ZFYVE28* | 0.000225 | 0.333142 |  |
| 4 | *LOC102399431* | 0.00023 | 0.333142 |  |
| 13 | *LOC112578579* | 7.06×10^-07^ | 0.019042 | DPR_NOCV |
| 13 | *CKAP2* | 3.72×10^-06^ | 0.050105 |  |
| 6 | *LOC123333976* | 8.67×10^-06^ | 0.05636 |  |
| 2 | *MBD5* | 9.16×10^-06^ | 0.05636 |  |
| 2 | *EPC2* | 1.05×10^-05^ | 0.05636 |  |
| 1 | *LOC102398510* | 2.02×10^-05^ | 0.090957 |  |
| 15 | *TMEM68* | 3.87×10^-05^ | 0.135484 |  |
| 15 | *LOC123329428* | 4.63×10^-05^ | 0.135484 |  |
| 4 | *NUP37* | 5.35×10^-05^ | 0.135484 |  |
| 6 | *CRTC2* | 5.35×10^-05^ | 0.135484 |  |

*Note: Chr – Chromosome no., FDR – False discovery rate corrected P-value, EN_NOCV – Elastic Net model without cross-validation, EN_CV – Elastic Net model with cross-validation, and DPR_NOCV – DPR model without cross-validation*

**Table S6: Top 10 genes and SNPs having lowest *P*-value in TWAS and GWAS with the SNF%, respectively**

| **Chr** | **SNP/Gene Name** | ***P*-value** | **FDR** | **Method** |
| --- | --- | --- | --- | --- |
| 10 | NC_037554.1:24182429[MU] | 2.38×10^-06^ | 0.09288 | GWAS |
| 3 | NC_037547.1:47608245[MU] | 7.42×10^-06^ | 0.1304 |  |
| 23 | NC_037567.1:41123236[MU] | 1.27×10^-05^ | 0.1304 |  |
| 11 | NC_037555.1:35249431[MU] | 1.34×10^-05^ | 0.1304 |  |
| 20 | NC_037564.1:45882062[MU] | 1.67×10^-05^ | 0.1304 |  |
| 3 | NC_037547.1:66715572[MU] | 2.11×10^-05^ | 0.1371 |  |
| 14 | NC_037558.1:54067539[MU] | 2.46×10^-05^ | 0.1371 |  |
| 13 | NC_037557.1:12532412[MU] | 3.00×10^-05^ | 0.1397 |  |
| 16 | NC_037560.1:10198062[MU] | 3.34×10^-05^ | 0.1397 |  |
| 9 | NC_037553.1:96723520[MU] | 3.77×10^-05^ | 0.1397 |  |
| 2 | *RFTN2* | 6.62×10^-07^ | 0.015164 | EN_NOCV |
| 10 | *LOC112587322* | 4.42×10^-06^ | 0.05067 |  |
| 21 | *DALRD3* | 7.91×10^-06^ | 0.060218 |  |
| 23 | *LOC112581526* | 1.05×10^-05^ | 0.060218 |  |
| 20 | *FAM174B* | 1.51×10^-05^ | 0.068908 |  |
| 9 | *LOC112587184* | 2.66×10^-05^ | 0.068908 |  |
| 4 | *LOC123465665* | 2.74×10^-05^ | 0.068908 |  |
| 2 | *RAB44* | 2.87×10^-05^ | 0.068908 |  |
| 9 | *LOC112587074* | 3.09×10^-05^ | 0.068908 |  |
| 9 | *RPL18A* | 3.61×10^-05^ | 0.068908 |  |
| 2 | *LHFPL5* | 7.25×10^-07^ | 0.016015 | EN_CV |
| 9 | *LOC112587092* | 2.90×10^-06^ | 0.032064 |  |
| 9 | *RDH8* | 8.10×10^-06^ | 0.059618 |  |
| 25 | *CDKL5* | 1.51×10^-05^ | 0.060046 |  |
| 24 | *LOC123465345* | 2.04×10^-05^ | 0.060046 |  |
| 5 | *RO60* | 2.32×10^-05^ | 0.060046 |  |
| 9 | *LOC112587184* | 2.64×10^-05^ | 0.060046 |  |
| 3 | *KPNA2* | 2.78×10^-05^ | 0.060046 |  |
| 16 | *LOC102410802* | 2.83×10^-05^ | 0.060046 |  |
| 2 | *RAB44* | 2.88×10^-05^ | 0.060046 |  |
| 2 | *CASP8* | 4.12×10^-06^ | 0.038007 | DPR_NOCV |
| 9 | *LOC123335169* | 4.17×10^-06^ | 0.038007 |  |
| 15 | *TRNAR-CCU_30* | 6.90×10^-06^ | 0.038007 |  |
| 9 | *ICAM3* | 7.94×10^-06^ | 0.038007 |  |
| 9 | *S1PR5* | 9.26×10^-06^ | 0.038007 |  |
| 9 | *LOC102410938* | 1.10×10^-05^ | 0.038007 |  |
| 6 | *EFCAB14* | 1.11×10^-05^ | 0.038007 |  |
| 24 | *LOC112581777* | 1.14×10^-05^ | 0.038007 |  |
| 5 | *LOC112585085* | 1.61×10^-05^ | 0.038007 |  |
| 17 | *GCN1* | 1.80×10^-05^ | 0.038007 |  |

*Note: Chr – Chromosome no., FDR – False discovery rate corrected P-value, EN_NOCV – Elastic Net model without cross-validation, EN_CV – Elastic Net model with cross-validation, and DPR_NOCV – DPR model without cross-validation*

**Table S7: Important SNPs for milk production identified based on their effect size on the TWAS genes**

| **snpID** | **MAF** | **ES** | **Gene** |
| --- | --- | --- | --- |
| NC_059180.1:39042759:A:C | 0.3125 | 0.003465 | *ADCY9* |
| NC_059180.1:37954703:T:G | 0.25 | 0.00277 |  |
| NC_059180.1:39042806:C:T | 0.3125 | -0.00379 |  |
| NC_059180.1:39360770:C:T | 0.5 | -0.00315 |  |
| NC_059165.1:91862476:C:T | 0.25 | 0.002359 | *CREB3L3* |
| NC_059165.1:90358674:T:C | 0.0625 | 0.001736 |  |
| NC_059165.1:91198615:G:A | 0.5 | -0.00278 |  |
| NC_059165.1:91951389:T:C | 0.75 | -0.00215 |  |
| NC_059171.1:81005196:C:G | 0.0625 | 0.39547 | *DGAT1* |
| NC_059171.1:81005040:A:G | 0.0625 | 0.393905 |  |
| NC_059171.1:81004959:T:C | 0.125 | -0.17715 |  |
| NC_059171.1:81004960:G:A | 0.125 | -0.17534 |  |
| NC_059171.1:25472680:C:T | 0.071429 | 0.36674 | *EIF3E* |
| NC_059171.1:26420896:T:C | 0.0625 | 0.268694 |  |
| NC_059171.1:26634012:G:A | 0.1875 | -0.1586 |  |
| NC_059171.1:26675575:T:G | 0.1875 | -0.15828 |  |
| NC_059165.1:69600024:C:A | 0.125 | 0.000162 | *LOC102411990* |
| NC_059165.1:69600051:A:G | 0.125 | 0.000107 |  |
| NC_059165.1:70794855:A:G | 0.0625 | -0.00015 |  |
| NC_059165.1:69600035:T:C | 0.125 | -6.37×10^-05^ |  |
| NC_059171.1:81711483:T:C | 0.1875 | 0.005846 | *LOC112579064* |
| NC_059171.1:81164256:C:T | 0.3125 | 0.005616 |  |
| NC_059171.1:81005049:A:G | 0.0625 | -0.00384 |  |
| NC_059171.1:81005196:C:G | 0.0625 | -0.00383 |  |
| NC_059173.1:44848478:A:G | 0.1875 | 0.008508 | *LOC112579963* |
| NC_059173.1:45127912:G:A | 0.125 | 0.006731 |  |
| NC_059173.1:45348489:A:G | 0.375 | -0.00272 |  |
| NC_059173.1:44848437:C:G | 0.0625 | -0.00253 |  |
| NC_059158.1:18094018:G:A | 0.4375 | 0.004523 | *LOC112583021* |
| NC_059158.1:18024718:T:C | 0.75 | 0.004109 |  |
| NC_059158.1:18234269:G:A | 0.3125 | -0.0084 |  |
| NC_059158.1:18234252:G:A | 0.4375 | -0.00729 |  |
| NC_059166.1:5233543:G:A | 0.125 | 0.001831 | *LOC112587322* |
| NC_059166.1:4838566:G:A | 0.125 | 0.001812 |  |
| NC_059166.1:4013048:T:C | 0.5 | -0.00232 |  |
| NC_059166.1:4358043:C:G | 0.3125 | -0.00148 |  |
| NC_059165.1:91315472:G:A | 0.0625 | 0.323986 | *MAP2K2* |
| NC_059165.1:91315538:C:T | 0.0625 | 0.317316 |  |
| NC_059165.1:91125451:C:T | 0.0625 | -0.52342 |  |
| NC_059165.1:91881413:C:A | 0.0625 | -0.50758 |  |
| NC_059165.1:95041857:G:A | 0.25 | 0.229646 | *MAP2K7* |
| NC_059165.1:93718046:T:A | 0.0625 | 0.224261 |  |
| NC_059165.1:94832544:A:G | 0.4375 | -0.6097 |  |
| NC_059165.1:94569021:G:C | 0.5 | -0.37786 |  |
| NC_059162.1:1113440:G:A | 0.3125 | 0.263725 | *MPZL1* |
| NC_059162.1:1235828:C:G | 0.125 | 0.202322 |  |
| NC_059162.1:988433:C:T | 0.1875 | -0.25521 |  |
| NC_059162.1:1113304:T:C | 0.5 | -0.069 |  |
| NC_059180.1:37211375:C:T | 0.0625 | 0.039745 | *NAGPA* |
| NC_059180.1:37211367:G:A | 0.0625 | 0.039659 |  |
| NC_059180.1:37211264:C:T | 0.125 | -0.03443 |  |
| NC_059180.1:37369488:T:C | 0.6875 | -0.02292 |  |
| NC_059165.1:92228259:C:T | 0.0625 | 0.077248 | *NDUFA11* |
| NC_059165.1:93684157:T:C | 0.0625 | 0.074948 |  |
| NC_059165.1:91985822:C:T | 0.5625 | -0.04801 |  |
| NC_059165.1:93139340:T:A | 0.1875 | -0.0442 |  |
| NC_059176.1:11197627:G:A | 0.5 | 0.238929 | *PPP4R4* |
| NC_059176.1:12449066:C:G | 0.0625 | 0.138531 |  |
| NC_059176.1:11808612:C:G | 0.0625 | -0.07244 |  |
| NC_059176.1:11725419:A:G | 0.1875 | -0.05992 |  |
| NC_059159.1:139715598:A:G | 0.0625 | 0.000119 | *TRNAW-CCA_36* |
| NC_059159.1:139813466:T:C | 0.071429 | 9.50×10^-05^ |  |
| NC_059159.1:140381399:C:A | 0.4375 | -0.0001 |  |
| NC_059159.1:140355477:G:A | 0.125 | -9.10×10^-05^ |  |

*Note: In the column snpID, the SNP numbers are represented as [chromosome_contig:Base_pair_pos:Reference_allele:Alternate_allele], MAF: minor allele frequency of the alternate allele, ES: effect size of the SNPs calculated from the SNP based gene prediction model via the DPR method.*

**Table S8: Important SNPs identified for fat percentage based on their effect size on the TWAS genes**

| **snpID** | **MAF** | **ES** | **Gene** |
| --- | --- | --- | --- |
| NC_059169.1:71396094:G:A | 0.5 | 0.264124 | *CAB39L* |
| NC_059169.1:71105614:C:T | 0.3125 | 0.096854 |  |
| NC_059169.1:71373709:G:A | 0.25 | -0.09493 |  |
| NC_059169.1:70996937:G:A | 0.0625 | -0.04007 |  |
| NC_059169.1:64751204:G:A | 0.375 | 0.001719 | *CCNA1* |
| NC_059169.1:64124633:T:C | 0.3125 | 0.001611 |  |
| NC_059169.1:64706472:G:A | 0.375 | -0.0014 |  |
| NC_059169.1:65394725:G:A | 0.3125 | -0.00129 |  |
| NC_059169.1:67843356:C:G | 0.125 | 0.106783 | *CKAP2* |
| NC_059169.1:68963140:C:T | 0.125 | 0.103494 |  |
| NC_059169.1:69017978:C:T | 0.875 | -0.10731 |  |
| NC_059169.1:67341678:C:T | 0.125 | -0.09983 |  |
| NC_059162.1:15876715:G:A | 0.3125 | 0.004564 | *CREB3L4* |
| NC_059162.1:15876581:A:T | 0.3125 | 0.004449 |  |
| NC_059162.1:15612607:G:A | 0.75 | -0.00474 |  |
| NC_059162.1:15929080:T:C | 0.5 | -0.00287 |  |
| NC_059162.1:17097030:T:C | 0.4375 | 0.395777 | *CRTC2* |
| NC_059162.1:15929080:T:C | 0.5 | 0.269177 |  |
| NC_059162.1:15876543:T:C | 0.0625 | -0.62491 |  |
| NC_059162.1:15876551:T:C | 0.0625 | -0.61613 |  |
| NC_059169.1:71149301:C:T | 0.4375 | 0.194809 | *EBPL* |
| NC_059169.1:70955459:T:A | 0.25 | 0.19063 |  |
| NC_059169.1:71149255:T:C | 0.0625 | -0.13581 |  |
| NC_059169.1:70996937:G:A | 0.0625 | -0.13439 |  |
| NC_059169.1:66976684:C:T | 0.1875 | 0.001426 | *FOXO1* |
| NC_059169.1:66976651:G:A | 0.1875 | 0.001404 |  |
| NC_059169.1:67248709:T:C | 0.3125 | -0.00134 |  |
| NC_059169.1:66996792:G:A | 0.25 | -0.00091 |  |
| NC_059169.1:34869710:G:A | 0.3125 | 4.07×10^-05^ | *LOC112578579* |
| NC_059169.1:35016020:G:A | 0.0625 | 2.97×10^-05^ |  |
| NC_059169.1:35015910:G:A | 0.125 | -3.70×10^-05^ |  |
| NC_059169.1:34874070:G:A | 0.25 | -3.57×10^-05^ |  |
| NC_059162.1:16540298:A:G | 0.0625 | 0.007586 | *LOC123333976* |
| NC_059162.1:15929217:T:A | 0.25 | 0.007334 |  |
| NC_059162.1:17096956:C:T | 0.125 | -0.01012 |  |
| NC_059162.1:15876551:T:C | 0.0625 | -0.00931 |  |

*Note: In the column snpID, the SNP numbers are represented as [chromosome_contig:Base_pair_pos:Reference_allele:Alternate_allele], MAF: minor allele frequency of the alternate allele, ES: effect size of the SNPs calculated from the SNP based gene prediction model via the DPR method.*

**Table S9: Important SNPs identified for SNF percentage based on their effect size on the TWAS genes**

| **snpID** | **MAF** | **ES** | **Gene** |
| --- | --- | --- | --- |
| NC_059158.1:140866608:C:T | 0.4375 | 1.855052 | *AOX1* |
| NC_059158.1:141518342:G:A | 0.3125 | 0.335252 |  |
| NC_059158.1:140509835:G:A | 0.1875 | -0.25926 |  |
| NC_059158.1:141445967:C:A | 0.125 | -0.19064 |  |
| NC_059158.1:141284199:G:A | 0.3125 | 0.195818 | *CASP8* |
| NC_059158.1:141518342:G:A | 0.3125 | 0.137084 |  |
| NC_059158.1:141038357:G:T | 0.1875 | -0.19597 |  |
| NC_059158.1:141445967:C:A | 0.125 | -0.10867 |  |
| NC_059173.1:10725250:T:C | 0.3125 | 0.315105 | *GCN1* |
| NC_059173.1:9935849:G:A | 0.25 | 0.140716 |  |
| NC_059173.1:10966760:G:A | 0.125 | -0.1841 |  |
| NC_059173.1:9577200:G:A | 0.0625 | -0.18222 |  |
| NC_059165.1:99908839:A:G | 0.0625 | 0.250446 | *IL27RA* |
| NC_059165.1:98738192:G:A | 0.0625 | 0.249902 |  |
| NC_059165.1:99132460:A:G | 0.3125 | -0.12927 |  |
| NC_059165.1:99406409:A:G | 0.25 | -0.0774 |  |
| NC_059165.1:99744965:G:A | 0.25 | 0.011715 | *LOC123335169* |
| NC_059165.1:99660240:C:T | 0.125 | 0.006962 |  |
| NC_059165.1:99660170:G:A | 0.25 | -0.00433 |  |
| NC_059165.1:99863264:C:T | 0.125 | -0.00317 |  |
| NC_059173.1:11521237:T:C | 0.0625 | 0.344932 | *PTPN11* |
| NC_059173.1:10966822:C:T | 0.0625 | 0.338652 |  |
| NC_059173.1:11571639:T:C | 0.0625 | -0.0851 |  |
| NC_059173.1:11571705:A:G | 0.0625 | -0.08428 |  |
| NC_059158.1:41058186:G:A | 0.1875 | 0.001236 | *RAB44* |
| NC_059158.1:41058155:A:C | 0.1875 | 0.001208 |  |
| NC_059158.1:41622083:T:C | 0.3125 | -0.00066 |  |
| NC_059158.1:41510663:G:A | 0.125 | -0.00057 |  |
| NC_059173.1:12439465:C:A | 0.5 | 0.004585 | *SDS* |
| NC_059173.1:11961647:C:T | 0.5625 | 0.002306 |  |
| NC_059173.1:10738179:A:G | 0.5625 | -0.00323 |  |
| NC_059173.1:10673891:T:C | 0.3125 | -0.00275 |  |
| NC_059173.1:12628699:A:C | 0.125 | 0.000928 | *SDSL* |
| NC_059173.1:10942288:C:G | 0.4375 | 0.000916 |  |
| NC_059173.1:12356788:C:T | 0.5 | -0.00181 |  |
| NC_059173.1:11961647:C:T | 0.5625 | -0.00102 |  |
| NC_059165.1:96611947:T:C | 0.125 | 0.100224 | *TYK2* |
| NC_059165.1:96311818:C:T | 0.5 | 0.083978 |  |
| NC_059165.1:96731859:G:A | 0.5625 | -0.36313 |  |
| NC_059165.1:96731721:A:G | 0.125 | -0.09184 |  |

*Note: In the column snpID, the SNP numbers are represented as [chromosome_contig:Base_pair_pos:Reference_allele:Alternate_allele], MAF: minor allele frequency of the alternate allele, ES: effect size of the SNPs calculated from the SNP based gene prediction model via the DPR method.*

**
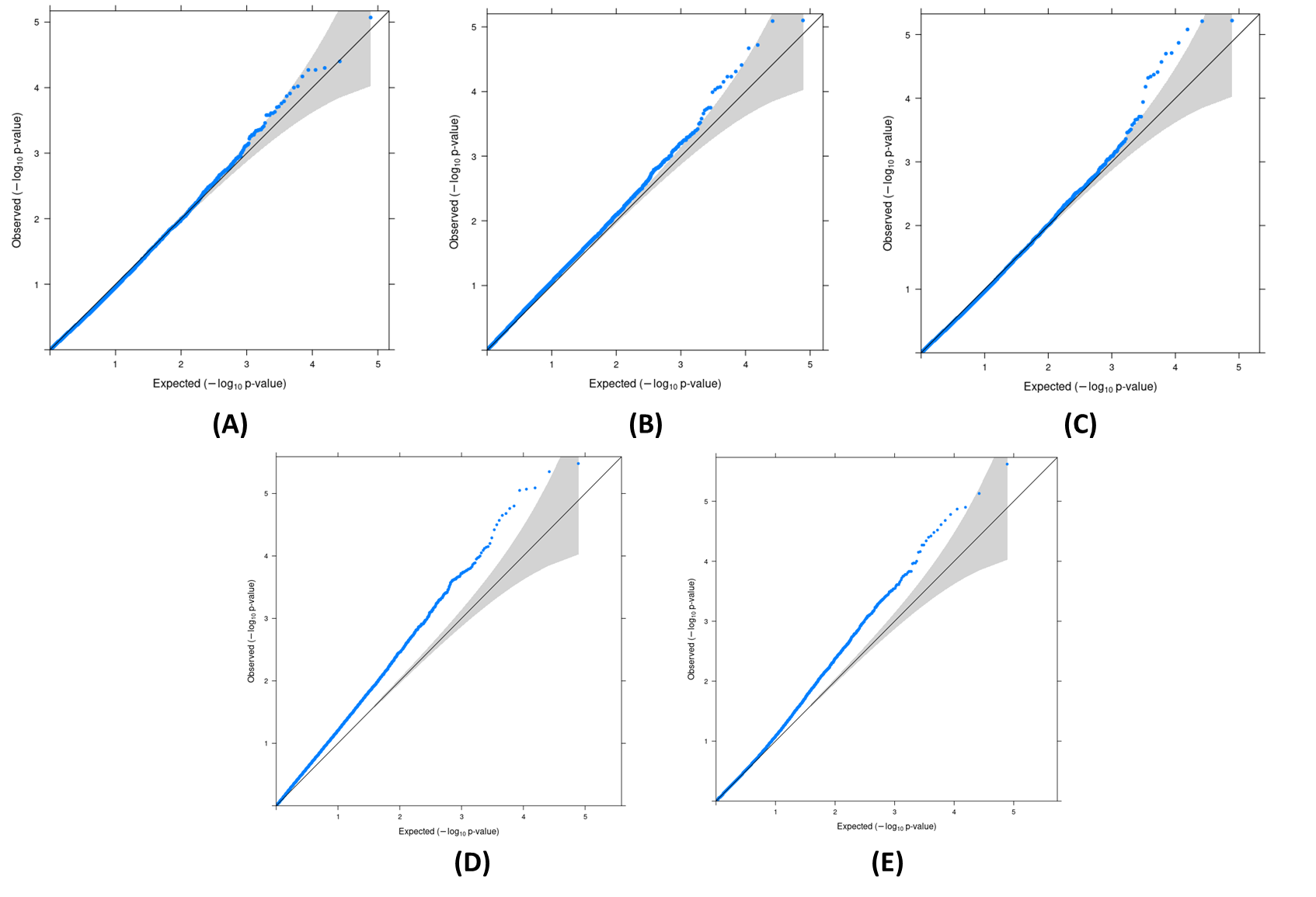
Fig. S1: Q-Q plot of distribution of the *P*-values in Genome-wide association study for (A) 305 DMY, (B) PY, (C) WA, (D) Fat%, and (E) SNF%**
